# Supported Vanadium Carbide Catalysts for Reverse Water Gas Shift and Methanol Steam Reforming: Activity, Stability, and Coking Pathways

**DOI:** 10.1021/acsami.5c16601

**Published:** 2025-11-21

**Authors:** Arturo Pajares, Sai Sharath Yadavalli, Hector Prats, Pilar Ramírez de la Piscina, Michail Stamatakis, Narcís Homs

**Affiliations:** † Materials & Chemistry, 54520 Flemish Institute for Technological Research (VITO NV), Boeretang 200, 2400 Mol, Belgium; ‡ Department of Chemistry, Inorganic Chemistry Laboratory, 6396 University of Oxford, South Parks Road, Oxford OX1 3QZ, U.K.; § Institute of Materials Chemistry, Technische Universität Wien, Getreidemarkt 9/165, 1060 Vienna, Austria; ∥ Departament de Química Inorgànica i Orgànica, secció de Química Inorgànica & Institut de Nanociència i Nanotecnología (IN2UB), 16724 Universitat de Barcelona, Martí i Franquès 1-11, 08028 Barcelona, Spain; ⊥ Catalonia Institute for Energy Research (IREC), Jardins de les Dones de Negre 1, 08930 Barcelona, Spain

**Keywords:** vanadium carbide, coking, transition metal carbides, CO_2_ conversion, methanol conversion

## Abstract

A series of supported vanadium carbide (VC_*x*_) catalysts were prepared, characterized, and tested for the carbon dioxide and methanol activation via the Reverse Water Gas Shift (RWGS) and Methanol Steam Reforming (MSR) reactions, respectively. Crystallite sizes of VC_*x*_ ranging from 9 to 36 nm were obtained depending on the support used (γ-Al_2_O_3_, SiO_2_, CeO_2_, ZrO_2_ and TiO_2_). In both reactions, the supported catalysts exhibited superior performance compared to the bulk VC_*x*_ sample. In the RWGS reaction, all catalysts showed high CO selectivity, with VC_*x*_/Al_2_O_3_ demonstrating the best performance and no significant deactivation after 100 h at 873 K. Under MSR conditions, VC_*x*_/ZrO_2_ achieved the highest methanol conversion. However, all catalysts suffered from significant deactivation due to coke formation, with CH_4_ as the main product instead of the desired H_2_ and CO_2_ from full steam reforming. Density Functional Theory (DFT) calculations revealed that methanol decomposition is more facile than CO_2_ decomposition on both stoichiometric VC and carbon-deficient V_8_C_7_ surfaces, particularly in the presence of carbon vacancies, leading to coke formation in the form of partially hydrogenated C_*x*_H_y_* species. These findings indicate that VC_*x*_ catalysts are more susceptible to coking under MSR than RWGS conditions, in line with experimental observations, and highlight the critical role of the carbide surface structure and vacancy concentration in coke formation.

## Graphical abstract

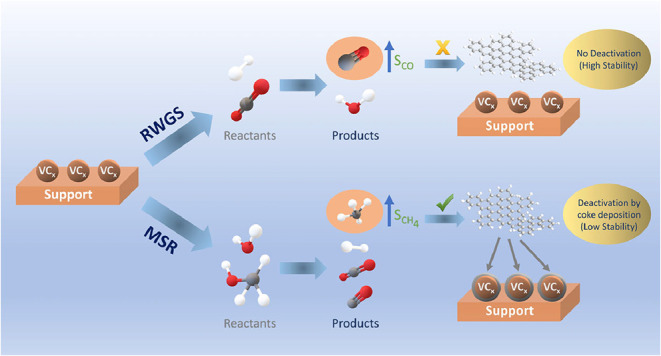

## 1 Introduction

Transition metal carbides (TMCs), particularly those derived from early transition metals in Groups 4–6, have attracted significant attention for their distinctive catalytic properties.^1^ Since the discovery of the Pt-like behavior of tungsten carbide (WC),^2^ TMCs have been extensively investigated in the fields of surface science and catalysis.^1,3–6^ These materials have emerged as promising, earth-abundant, and cost-effective alternatives to the precious noble metals traditionally used in several catalytic reactions. This is largely due to their similar electronic structures, excellent chemical and physical resistance, and high tolerance to coking and sulfur poisoning, all of which enhance their durability in catalytic processes.^1,7,8^

While molybdenum and tungsten carbide-based catalysts have been widely studied,^9–11^ vanadium carbide (VC_*x*_) catalysts remain relatively unexplored. Single-crystal studies have shown that stoichiometric vanadium carbide (VC) enhances C–H bond activation in alkanes, while interacting less strongly with C=C bonds in alkenes compared to metallic vanadium.^12^ This unique reactivity is similar to that of Pt-group metals and is also observed for Mo_2_C and WC. Notably, VC_*x*_ catalysts have been demonstrated to be catalytically active for several reactions, including ammonia decomposition, oxygen reduction reaction (ORR), hydrogen evolution reaction (HER), and *n*-butane dehydrogenation.^13–18^

In previous work, we highlighted the key role of carbon vacancies in VC_*x*_ during the Reverse Water Gas Shift (RWGS) reaction (CO_2_ + H_2_ → CO + H_2_O; Δ_r_ *H*° = 41.2 kJ mol^–1^ at 298 K), where they enhance both activity and CO selectivity, while suppressing side reactions such as CO_2_ and CO methanation.^19^ Density Functional Theory (DFT) calculations further revealed that the carbon-deficient V_8_C_7_ phase adsorbs both CO_2_ and H_2_ more strongly and facilitates their dissociation, lowering the CO_2_ dissociation barrier from 1.53 eV on VC to 0.62 eV on V_8_C_7_, and the H_2_ dissociation barrier from 0.65 eV on VC to 0.16 eV on V_8_C_7_.^19,20^ Additionally, studies of Al_2_O_3_-supported VC_*x*_ nanoparticles demonstrated that the presence of carbon vacancies and large VC_*x*_-Al_2_O_3_ interfacial areas are beneficial for the RWGS reaction.^21^ These supported catalysts exhibited only minor deactivation under reaction conditions.^19,21^

In contrast, under methanol steam reforming (MSR) reaction conditions, we revealed that bulk VC_*x*_ is highly selective toward CH_4_ formation instead of H_2_ + CO_2_ (CH_3_OH + H_2_O → CO_2_ + 3H_2_; Δ_r_ *H*° = 49.7 kJ mol^–1^ at 298 K)^22–25^ and undergoes severe deactivation due to carbon deposition.^22^ This high selectivity toward CH_4_ has also been observed on stoichiometric VC single crystals, suggesting that the complete decomposition of methanol (CH_3_OH) proceeds through C–O bond cleavage of intermediate methoxy species, yielding CH_4_ and surface O.^26^

Building on this background, we have prepared, characterized, and evaluated a series of VC_*x*_-based catalysts supported on various oxides (γ-Al_2_O_3_, SiO_2_, CeO_2_, ZrO_2_ and TiO_2_) for both the RWGS and MSR reactions as case studies, with the aim of investigating their catalytic behavior, stability and deactivation pathways. We show how the structural features of the supported VC_*x*_ nanoparticles correlate with their catalytic behavior under these conditions. In addition, we conduct DFT calculations to elucidate and compare the coke-formation pathways for both RWGS and MSR reactions on VC and V_8_C_7_ surface models. This combined experimental and theoretical work aims to provide new insights into the structure–reactivity relationships and coking mechanisms of vanadium carbide catalysts in industrially relevant processes.

## 2 Methodology

### 2.1 Preparation

γ-Al_2_O_3_ (*Alfa Aesar*, 22*6 m* ^2^ *g* ^*–1*^), SiO_2_ (*Degussa*, 2*00 m* ^2^ *g* ^*–1*^), CeO_2_ (*Tecnan*, *90 m* ^2^ *g* ^*–1*^), ZrO_2_ (*Tecnan*, *50 m* ^2^ *g* ^*–1*^) and TiO_2_ (*Tecnan*, *117 m* ^2^ *g* ^*–1*^ *anatase/rutile = 78/22 wt %/wt*) were used as support materials. The supports were first immersed in 50 mL of ethanol to form a suspension. Then, an equimolar amount of vanadium oxytriisopropoxide *(VO­(isopropoxide)*_3_, *Alfa Aesar 96%)* and 4,5-dicyanoimidazole (*C*_5_ *H*_2_ *N*_4_, *Manchester Organics 97%*) was added to yield ∼25 wt % of V in the final catalyst, based on procedures previously reported for the preparation of bulk and alumina-supported VC_*x*_ catalysts.^19,21,22^ The suspension was stirred at room temperature under Ar until the ethanol had evaporated. Afterward, the solid was treated in a tubular furnace under a flow of Ar up to 1373 K (2.5 K min^–1^) for 5 h and then cooled down. The catalysts were named VC_*x*_/Al_2_O_3_, VC_*x*_/SiO_2_, VC_*x*_/CeO_2_, VC_*x*_/ZrO_2_ and VC_*x*_/TiO_2_ indicating the support used in the preparation. For comparison, a bulk VC_*x*_ sample was prepared following the same procedure but in the absence of support.

### 2.2 Characterization

The crystal structures of the samples were characterized by X-ray diffraction (XRD) analysis with a PANalytical X’Pert PRO MPD Alpha1 powder diffractometer, using a Cu Kα radiation source (λ = 1.5406 Å). The crystallite size was calculated using the Debye–Scherrer equation. Nitrogen adsorption–desorption was performed at 77 K, using a Micromeritics Tristar II 3020 instrument. Before the measurement, the catalyst was degassed at 525 K for 5 h under N_2_. The pore size distribution was determined by applying the Barrett–Joyner–Halenda (BJH) method.

Inductively coupled plasma atomic emission spectrometry (ICP-AES) was used for the analysis of the chemical composition of the catalysts. The ICP-AES measurements were carried out using a PerkinElmer Optima 3200RL apparatus. X-ray photoelectron spectroscopy (XPS, PerkinElmer PHI-5500 Multitechnique System, Physical Electronics) was used to analyze the surface of the samples. All spectra were collected using an Al X-ray source (*h*ν = 1486.6 eV and 350 W). Calibration of the instrument was confirmed using Au as a reference and the binding energy (BE) of the C 1s peak at 284.8 eV.

Transmission electron microscopy (TEM) images were collected employing a JEOL J2010 F microscope operated at up to 200 kV. Energy dispersive X-ray (EDX) analysis was carried out in an Oxford instrument INCA *x*-sight. H_2_-temperature-programmed reduction (H_2_-TPR) experiments were performed in a Micromeritics Autochem II 2920 equipped with a thermal conductivity detector (TCD). Samples were pretreated at 363 K under He for 1 h and then exposed to an H_2_/Ar (12% v/v) flow, and the temperature was then increased up to 1073 at 10 K min^–1^.

Raman spectroscopy analysis was performed in a Jobin-Yvon LabRam HR 800 instrument, with an optical Olympus BXFM microscope with a 532 nm laser and a CCD detector. The laser power was restricted to 1.25 mW to avoid undesired laser-heating effects during spectra acquisition. Thermogravimetric analysis (TGA) was performed on a NETZSCH STA449 F3 Jupiter instrument coupled to a Pfeiffer mass spectrometer (MS). Samples were heated from 298 to 1073 K (5 K min^–1^) under air flow. MS signals were recorded during the analysis.

### 2.3 Catalytic Tests

#### 2.3.1 RWGS Reaction

The RWGS catalytic tests were performed in a Microactivity unit (PID Eng&Tech), using 300 mg of catalyst, a catalytic bed of 1 mL, and SiC as the diluting agent. The catalytic bed was loaded into a tubular reactor. The samples were heated under N_2_ flow from room temperature up to 525 K. Then, they were exposed to a reactant gas mixture of CO_2_/H_2_/N_2_ = 1/3/1 (mol/mol) at a constant gas hourly space velocity (GHSV) of 3000 h^–1^.

All catalysts were studied within a temperature range of 523 to 873 K at 0.1 MPa; the catalysts were kept for 2.5 h at each temperature, which was reached after 10 min of heating; the first measurement was taken after 30 min at a given temperature. A Varian 450-GC, equipped with a TCD and two FIDs, was used for the online analysis of the products. CO_2_ conversion and product distribution were determined at each temperature from the average of 4 measurements. The main products obtained were CO and CH_4_. The CO_2_ conversion (*X*_CO_2__) and the selectivity to carbon-product *i* (*S*_*i*_) were determined as follows (eqs 1 and 2)­

1 $$X_{{CO}_{2}}\;\left(\%\right)=\left(1-\frac{{\left(C_{{CO}_{2}}\right)}_{outlet}}{{\left(C_{{CO}_{2}}\right)}_{outlet}+\sum {\left(C_{i}\right)}_{outlet}}\right)\times 100$$

2 $$S_{i}\;\left(\%\right)=\frac{{\left(C_{i}\right)}_{\mathrm{outlet}}}{\sum {\left(C_{i}\right)}_{\mathrm{outlet}}}\times 100$$

where *C*_*i*_ and *C*_CO_2__ denote the molar concentrations of the *i* product (CH_4_ or CO) and CO_2_, respectively.

#### 2.3.2 MSR Reaction

The MSR catalytic tests were performed using a Microactivity-Reference unit (PID Eng&Tech), similar to the setup used for the RWGS tests described above, but equipped with a GILSON liquid pump for injecting the reactant mixture H_2_O/CH_3_OH = 1/1 (molar ratio) at a constant flow and at atmospheric pressure.

The preheated liquid mixture (473 K) was mixed with N_2_, H_2_O/CH_3_OH/N_2_ = 1/1/1.2 (molar ratio) and flowed through the catalytic bed. A liquid–gas separator working at 277 K allowed the condensation of vapors at the outlet of the system. A total of 300 mg of catalyst were diluted in a catalytic bed of 1 mL with SiC. All tests were carried out at 573–723 K, 0.1 MPa and a GHSV of 2500 h^–1^. Each reaction temperature was reached after 10 min of heating, and the first measurement was taken after 30 min at each temperature. The CH_3_OH conversion and product distribution were determined at each temperature from the average of 4 different measurements. The catalysts were kept for 1.5 h at each temperature. At 723 K, the catalysts were kept for 20 h under reaction to study the stability of these materials. The gaseous products were analyzed online employing a Varian 4900 micro-GC equipped with three channels with micro TCDs.

The CH_3_OH conversion (*X*_CH_3_OH_) was defined as follows (eq 3)­

3 $$X_{{\mathrm{CH}}_{3}\mathrm{OH}}\;\left(\%\right)=\frac{\sum a_{i}\cdot {{(\eta }_{i})}_{\mathrm{outlet}}}{{{(\eta }_{CH_{3}\mathrm{OH}})}_{\mathrm{inlet}}}\times 100$$

where *a*_*i*_ is the number of carbon atoms per molecule of the product *i* (CH_4_, CO, CO_2_, HCHO, C_2_H_4_ and C_2_H_6_), η_*i*_ is the number of moles of the product *i*, and η_CH_3_OH_ is the number of moles of methanol. The product distribution is given as molar fraction (*Y*_*i*_) of products obtained (eq 4)­

4 $$Y_{i}\;\left(\%\right)=\frac{{\left(C_{i}\right)}_{\mathrm{outlet}}}{\sum {\left(C_{i}\right)}_{\mathrm{outlet}}}\times 100$$

where *C*_*i*_ is the concentration of the product *i*.

### 2.4 Computational Methods

The *Vienna Ab Initio Simulation Package* (VASP, version 5.4.4) was employed to carry out periodic DFT calculations. The exchange-correlation effects were captured using the Perdew–Burke–Ernzerhof (PBE) functional together with the D3 dispersion correction.^27,28^ The effect of core electrons was approximated using the Projector Augmented Wave (PAW) method.^29,30^ All calculations used an energy cutoff of 415 eV and electronic and ionic convergence criteria of 10^–5^ eV and 10^–2^ eV Å^–1^, respectively.

The slab models for the stoichiometric (VC) and C-deficient (V_8_C_7_) phases were obtained from the corresponding optimized bulk structures. The VC slab consist of a 3 × 3 supercell, while the V_8_C_7_ slab used a 1 × 1 supercell, both including 4 layers with the two bottommost layers fixed to bulk positions (Figure S1). Note that the V_8_C_7_ phase consists of ordered vacancies and has been reported to be more stable than the stoichiometric VC phase.^31^ All slab calculations were carried out using a Γ-centered 5×5×1 *k*-point mesh. A vacuum layer of at least 18 Å was included to eliminate interactions between the periodic slab images, and dipole corrections were applied in the *z*-direction.

All transition states (TS) were located using the ML-NEB module included in CatLearn.^32^ A vibrational analysis was performed by means of the finite difference method using a displacement of 0.02 Å, in order to confirm the nature of each TS by verifying the presence of a single imaginary frequency. All free energy contributions were computed using the ideal-gas model for gas-phase molecules (including translations, rotations and vibrations as necessary for each species), and the harmonic oscillator model for the adsorbed species, as implemented in the ASE thermochemistry module.^33^

## 3 Results

### 3.1 Characterization of the Catalysts

Table 1 shows the vanadium content and *S*_BET_ values for the supported VC_*x*_ catalysts prepared using the procedures detailed in Section 2.1. The *S*_BET_ values obtained for all catalysts were in the range of 86–271 m^2^ g^–1^. The *S*_BET_ of VC_*x*_/Al_2_O_3_ and VC_*x*_/CeO_2_ decreased relative to their respective fresh supports (see Section 2.1 for *S*_BET_ of fresh supports). However, for VC_*x*_/SiO_2_, VC_*x*_/ZrO_2_ and VC_*x*_/TiO_2_, an increase in *S*_BET_ was observed. All catalysts were mainly mesoporous materials with average pore widths ranging over 6–17 nm (Table 1 and Figure S2).

**Table 1:** Several Characteristics of Supported VC_*x*_ Catalysts before and after RWGS and MSR Reaction

			*S*_BET_ (m^2^ g^–1^)			pore size (nm)		
catalyst	V content (wt %)	crystallite size of VC*_x_* from XRD (nm)	fresh	used RWGS	used MSR	fresh	used RWGS	used MSR
VC*_x_*/Al_2_O_3_	22.1	9	210	101	24	9	11	9
VC*_x_*/SiO_2_	21.3	9	216	92	37	7	9	10
VC*_x_*/CeO_2_	22.5	36	86	45	9	6	6	6
VC*_x_*/ZrO_2_	21.6	25	119	59	8	6	6	21
VC*_x_*/TiO_2_	23.0	20	158	82	48	17	18	16
VC*_x_*		11	271	219	5	7	10	35

The XRD peaks located at 2θ = 37.7, 43.4, 63.1, 75.6 and 79.7° correspond to reflections characteristic of cubic vanadium carbides, which may include V_8_C_7_ (JCPDS 35-0786) and VC (JCPDS 01-073-0476) (Figure 1). Because the diffraction patterns of these two phases are very similar, their distinction based solely on XRD is challenging. Additionally, no crystalline VO_*x*_ species were detected (Figure 1). In a prior study, we investigated VC_*x*_ samples synthesized using a similar procedure to the one employed in this current work, and found that the resulting bulk material consisted predominantly of V_8_C_7_ rather than stoichiometric VC, as confirmed by high-resolution STEM analysis.^19^ Given that the supported VC_*x*_ catalysts were synthesized following the same route, it can be inferred that they also contain a significant proportion of V_8_C_7_, while the fraction of stoichiometric VC likely increases with particle sintering.

**Figure 1:**
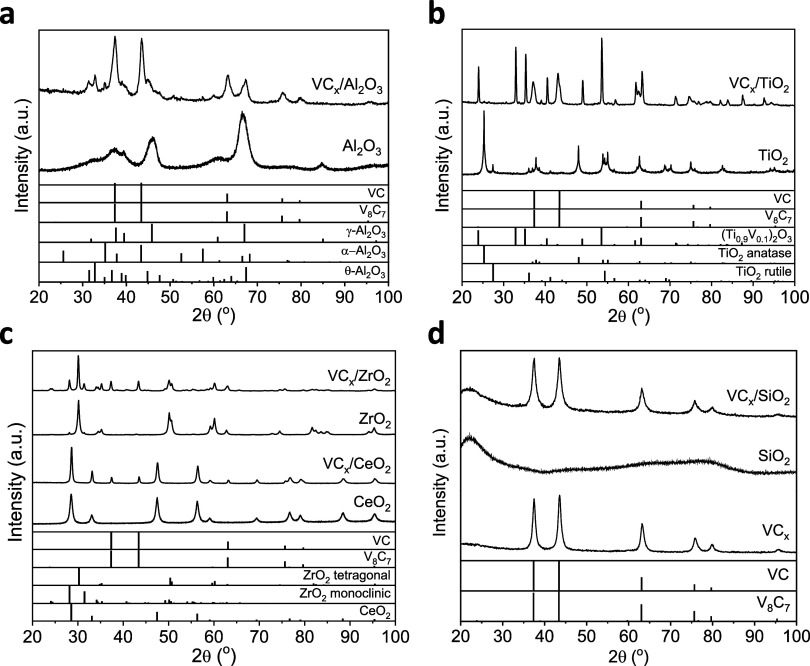
XRD patterns of the (a–d) supported and (d) bulk VC_*x*_ catalysts used in this work and their corresponding support materials.

During the preparation of supported catalysts, materials were treated at 1373 K, and some supports underwent structural changes. A partial transformation of γ-Al_2_O_3_ cubic phase (JCPDS 10-0425) into monoclinic (JCPDS 35-0121) and rhombohedral (JCPDS 10-0173) phases was detected, as a result of the high temperature employed during the preparation of VC_*x*_/Al_2_O_3_ (Figure 1a).^34,35^ For VC_*x*_/TiO_2_, the presence of (Ti_0.9_V_0.1_)_2_O_3_ rhombohedral solid solution (JCPDS 01-071-0275) is deduced (Figure 1b). In the case of ZrO_2_ with both tetragonal (JCPDS 50-1089) and monoclinic (JCPDS 37-1484) phases, a small increase in the amount of monoclinic phase is proposed to have occurred during the preparation of VC_*x*_/ZrO_2_ (Figure 1c). In VC_*x*_/CeO_2_, cubic CeO_2_ (JCPDS 34-0394) was found (Figure 1c). The crystallite sizes of VC_*x*_, measured by XRD for all catalysts, are listed in Table 1. Both VC_*x*_/Al_2_O_3_ and VC_*x*_/SiO_2_ catalysts exhibited slightly smaller sizes (9 nm) compared to the bulk VC_*x*_ catalyst (11 nm). Conversely, higher crystallite sizes were obtained for all the other catalysts (Table 1). This variation in VC_*x*_ crystallite size can be attributed to the surface area of the material support, as supports with higher *S*_BET_ values restrict the sintering of the VC_*x*_ particles during preparation, leading to the formation of smaller VC_*x*_ nanoparticles.

The samples exhibited Raman bands at 1350 and 1598 cm^–1^, which were attributed to residual carbon formed during the preparation process (Figure S3). Additionally, the Raman bands at 97, 143, 192, 285, 694, and 995 cm^–1^ could be associated with the presence of oxy-vanadium species.^36,37^ Furthermore, for VC_*x*_/CeO_2_, the band at 461 cm^–1^ is attributed to CeO_2_, and those at 122, 264, 375, 785, and 854 cm^–1^ to CeVO_4_.^38–40^ The poorly defined bands at 269, 413, and 603 cm^–1^ in the Raman spectrum of VC_*x*_/TiO_2_, are related to the presence of rutile TiO_2_.^41^

The TEM analyses confirmed the presence of smaller VC_*x*_ particles in VC_*x*_/Al_2_O_3_ (9.0 nm) and VC_*x*_/SiO_2_ (8.9 nm) compared to the other three catalysts (Figure S4), in accordance with XRD results. Furthermore, STEM-EDX analysis revealed a homogeneous distribution of V along the support in all cases. Characterization by HRTEM allowed the determination of *d*-spacings of 0.241 and 0.208 nm, assigned to the (222) and (400) facets of V_8_C_7_ or the (111) and (200) facets of stoichiometric VC, respectively.

H_2_-TPR experiments indicated, in all cases, a relatively small amount of H_2_ consumption (Table 2). In the case of VC_*x*_/Al_2_O_3_, VC_*x*_/ZrO_2_ and VC_*x*_/CeO_2_ samples, the H_2_ consumption peak at 732–750 K is attributed to the reduction of surface mono- or polymeric oxy-vanadium­(V) species (Figure 2a). If amorphous V_2_O_5_ had been formed, it would be reduced at about 852 K and in the case of crystalline V_2_O_5_ at even higher temperatures.^19,42–44^ The peak at 1010 K in the H_2_-TPR of VC_*x*_/CeO_2_ is related to the reduction of VCeO_4_.^45^ The presence of these species was previously determined by Raman analysis. The slight shifts in the reduction peaks can be attributed to the interaction of VO_*x*_ species formed on VC_*x*_ with the support.^46^ H_2_ consumption peaks centered at about 500–600 K in the profiles of VC_*x*_/SiO_2_ and bulk VC can be associated with the reduction of vanadium oxy-carbide species, as proposed in prior studies (Figure 2a).^19,22^ The presence of various species including oxy-carbide and oxy-vanadium species is related to the H_2_-consumption profile of VC_*x*_/TiO_2_ (Figure 2a).

**Figure 2:**
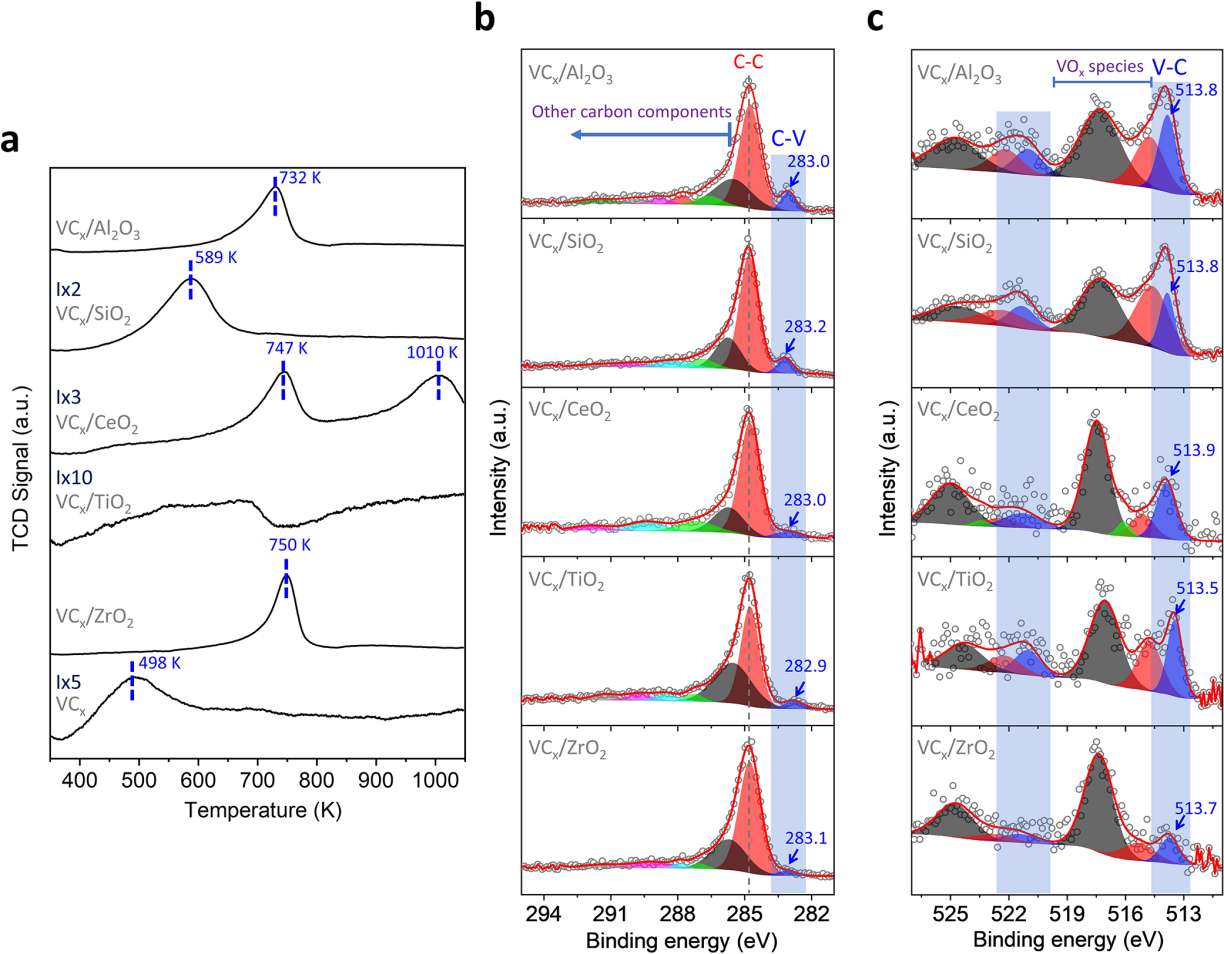
(a) H_2_-TPR profiles, (b) C 1s XPS levels, and (c) V 2p XPS levels of the supported VC_*x*_ catalysts.

**Table 2:** H_2_ Consumption Determined by TPR, V/M (mol/mol) Ratio from Chemical Analysis (ICP) and XPS, and Dispersion Factor Determined by XPS

			(V/M)_XPS_ (M = Al,Si,Ce,Zr,Ti)(mol/mol)		
catalyst	H_2_ consumption (molH_2_/molV)	(V/M)_ICP_ (M = Si,Ce,Zr,Al,Ti) (mol/mol)	fresh	used RWGS	dispersion factor^b^
VC*_x_*/Al_2_O_3_	0.198	0.388	0.735	0.648	1.894
VC*_x_*/SiO_2_	0.181	0.447	1.664	1.739	3.723
VC*_x_*/CeO_2_	0.050	2.714	2.654	3.139	0.978
VC*_x_*/ZrO_2_	0.160	0.862	1.242	1.801	1.441
VC_x_/TiO_2_	0.046	0.529	0.674	0.959	1.274
VC*_x_*	0.015^a^				

a Assuming 100% of VC on the sample.

b Calculated from the fresh catalysts.

XPS was used for the surface characterization of samples (Figure 2b,c and S5–S9). Specifically, the C *1s* core level spectra revealed bands at 282.9–283.2 eV, assigned to the carbide species, (VC_*x*_) (Figure 2b).^13,17–19,21,22^ The peak at 284.8 eV was attributed to adventitious carbon and C–C bonds belonging to residual carbon formed during the preparation process.^47^ Shoulders observed at BE higher than 284.8 eV were associated with C–O, C=O and O=C–O bonds.^48–50^ The V *2p* spectra were deconvoluted into 3 or 4 doublets (V *2p*_*3/2*_ – V *2p*_*1/2*_), depending on the sample (Figure 2c). Peaks at low BE, ranging from 513.5–514.0 eV, were attributed to VC_*x*_.^13,17–19,21,22^ V *2p*_*3/2*_ bands at higher BE are assigned to oxy-vanadium species.^13,17–19,21,22^ XPS spectra corresponding to O *1s* and characteristic bands of the supports, Al *2p*, Si *2p*, Ce *3d*, Ti *2p* and Zr *3d* (Figure S5–S9), are discussed in the Supporting Information.

Using the V/M mol/mol (M = Al, Si, Ce, Zr, Ti) determined by XPS and chemical analysis it is possible to calculate the dispersion factor (*D*) that provides a semiquantitative assessment of the vanadium carbide dispersion onto the support (eq 5)^51^

5 $$D=\frac{{\left(V/M\right)}_{\mathrm{XPS}}}{{\left(V/M\right)}_{\mathrm{ICP}}}$$

The dispersion factors obtained from both XPS and chemical analysis (ICP) are summarized in Table 2. VC_*x*_/SiO_2_ and VC_*x*_/Al_2_O_3_ exhibited the highest dispersion factors among all samples. This observation may be attributed to their elevated *S*_BET_ values and the small crystallite sizes of VC_*x*_ in these two samples.

### 3.2 RWGS Reaction

The catalytic behavior of the supported catalysts is depicted in Figure 3. The catalysts showed a high CO_2_ conversion ranging from 31 to 52% at 873 K (equilibrium conversion = 60% at 873 K) depending on the support used (Figure 3a,b). All catalysts exhibited increasing CO selectivity with temperature in the studied range (573–873 K), approaching 100% at 873 K (Figure 3c), with CH_4_ as the main byproduct. For VC_*x*_/TiO_2_, the anomalous profile of CO_2_ conversion as a function of temperature may be related to a structural transformation of the support during the catalytic test, as discussed later (Figure 3b). The bulk VC_*x*_ catalyst showed a higher CO_2_ conversion than the supported VC_*x*_ catalysts in the whole range of temperatures considered. However, supported catalysts produced a much higher amount of CO per mol of V (mol_CO_ mol_V_^–1^ h^–1^) when compared to bulk VC_*x*_ (Figure 3d).

**Figure 3:**
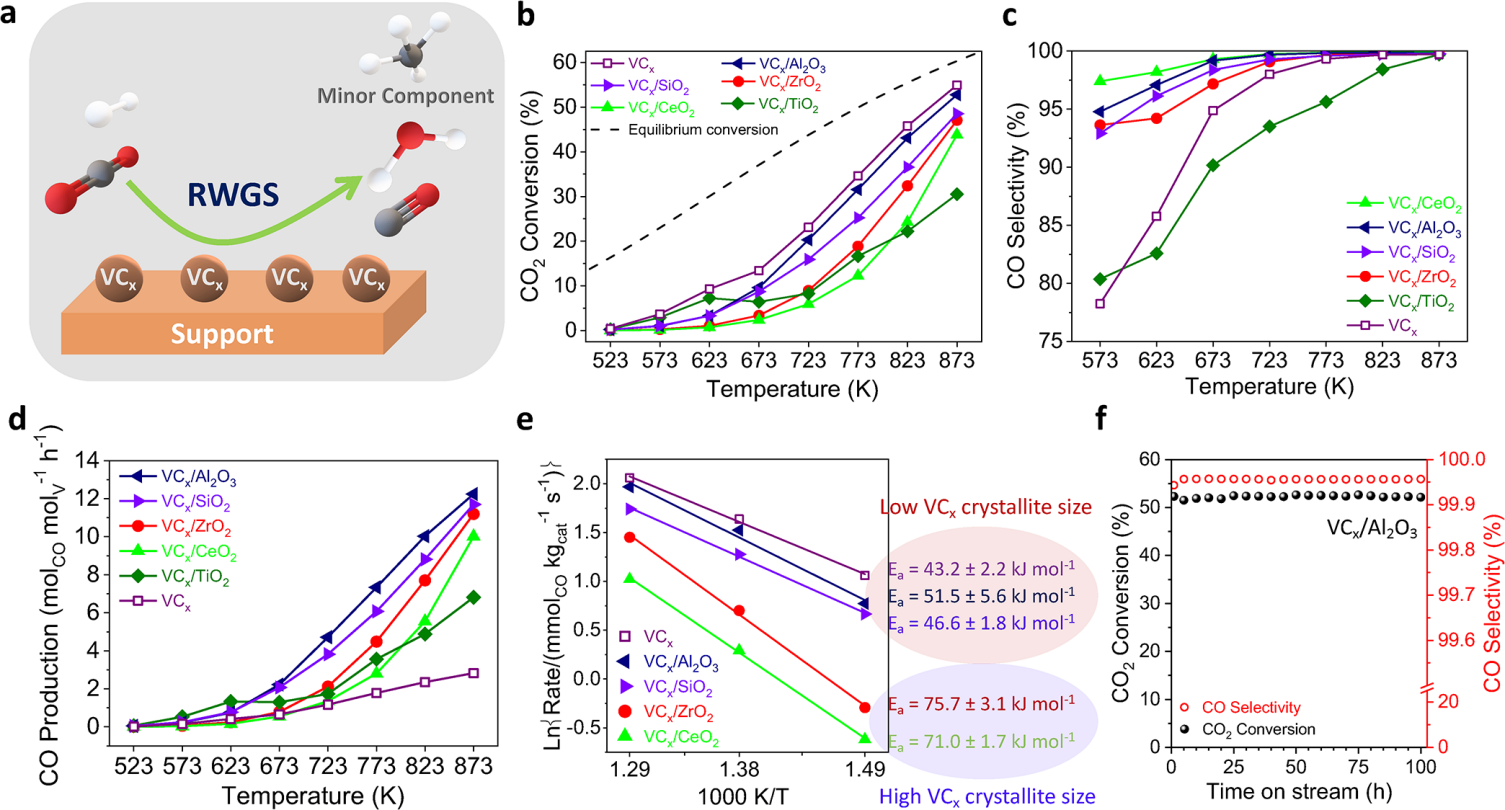
Catalytic behavior of supported VC_*x*_ catalysts in the RWGS reaction. (a) Schematic of the reaction, (b) CO_2_ conversion, (c) CO selectivity, (d) CO production, (e) apparent activation energy in the range of 673–773 K, and (f) stability test over VC_*x*_/Al_2_O_3_ at 873 K for 100 h. Reaction conditions: CO_2_/H_2_/N_2_ = 1/3/1; GHSV = 3000 h^–1^; *P* = 0.1 MPa; m = 300 mg.

Figure 3e depicts the Arrhenius plots and the apparent activation energies (*E*_a_) in the 673–773 K range for supported and bulk VC_*x*_ catalysts. The VC_*x*_/CeO_2_ and VC_*x*_/ZrO_2_ samples, with crystallite sizes of 36 and 25 nm, respectively, showed much higher *E*_a_ than VC_*x*_/Al_2_O_3_, VC_*x*_/SiO_2_, and bulk VC_*x*_, which had smaller crystallite sizes (9–11 nm) (Figure 3e and Table 1). The catalytic behavior of the supported VC_*x*_ samples in the RWGS reaction is largely governed by the structural characteristics of the carbide phase on each support. For catalysts containing small VC_*x*_ particles, a higher number of carbon vacancies is expected, consistent with previous observations where smaller VC_*x*_ crystallites exhibited predominantly C-deficient V_8_C_7_ domains rather than stoichiometric VC.^19^ The superior performance of VC_*x*_/Al_2_O_3_ and VC_*x*_/SiO_2_ is therefore attributed mainly to their higher density of carbon vacancies, which, as discussed earlier, enhance the adsorption of CO_2_ and H_2_ and lower the corresponding dissociation barriers.^19^ These results suggest that the active sites involved in CO_2_ and H_2_ activation are under-coordinated vanadium atoms located near carbon vacancies.

Additionally, the contribution of support-VC_*x*_ interfacial effects cannot be ruled out. For instance, in Mo_*x*_C/Al_2_O_3_ systems, the surface OH groups of Al_2_O_3_ have been shown to enhance the RWGS activity by facilitating CO activation through the formation of bicarbonate and formate intermediates at the carbide-support interface, ultimately promoting CO production.^52^

Overall, the VC_*x*_/Al_2_O_3_ catalyst displayed a CO_2_ conversion of 52% at 873 K with nearly 100% CO selectivity, matching or surpassing the activity of other non-noble-metal catalysts reported in the literature (Table S1). This result highlights VC_*x*_/Al_2_O_3_ as a cost-effective and earth-abundant alternative to traditional noble-metal systems for RWGS reaction.

The VC_*x*_ crystallization on the different supports is likely influenced by the interaction between the support and the sol–gel vanadium precursor. This interaction could determine the final VC_*x*_ crystallite size. Within this context, for Mo_2_C-based catalysts prepared by impregnation and subsequent carburization, a high influence of the support on their characteristics and performance in the RWGS has also been found.^53^

After RWGS catalytic tests, XRD patterns of VC_*x*_/Al_2_O_3_, VC_*x*_/SiO_2_, VC_*x*_/CeO_2_ and bulk VC_*x*_ catalysts were similar to those of the corresponding fresh ones, (Figures 4a and S10). However, for the other catalysts, some variations of the crystalline phases associated with the support were noticed. The presence of (Ti_0.9_V_0.1_)_2_O_3_ cannot be deduced from the XRD pattern of used VC_*x*_/TiO_2_, and an increase in the amount of anatase and rutile phases after the RWGS test can be deduced, compared to the fresh catalyst (Figure S10). For VC_*x*_/ZrO_2_, a partial transformation of tetragonal ZrO_2_ onto the monoclinic phase during the catalytic test can be proposed (Figure S10). In all cases, after the RWGS catalytic test, no variations in the crystallite sizes of VC_*x*_ were determined by XRD with respect to those of the fresh catalysts. Moreover, the presence of surface carbide species was determined in the used catalysts (Figures 4b,c and S6–S9); see C *1s and V 2p*_*3/2*_ at 282.8–283.1 and 513.7–514.0 eV, respectively, attributed to VC_*x*_. However, an increase in the amount of surface oxy-vanadium species took place during the RWGS test, as can be deduced from V *2p* spectra (Figures 4c and S6–S9). The Al *2p*, Si *2p*, Ce *3d*, Ti *2p* and Zr *3d* spectra characteristic of the different supports were also found for the postreaction catalysts (Figures S5–S9).

**Figure 4:**
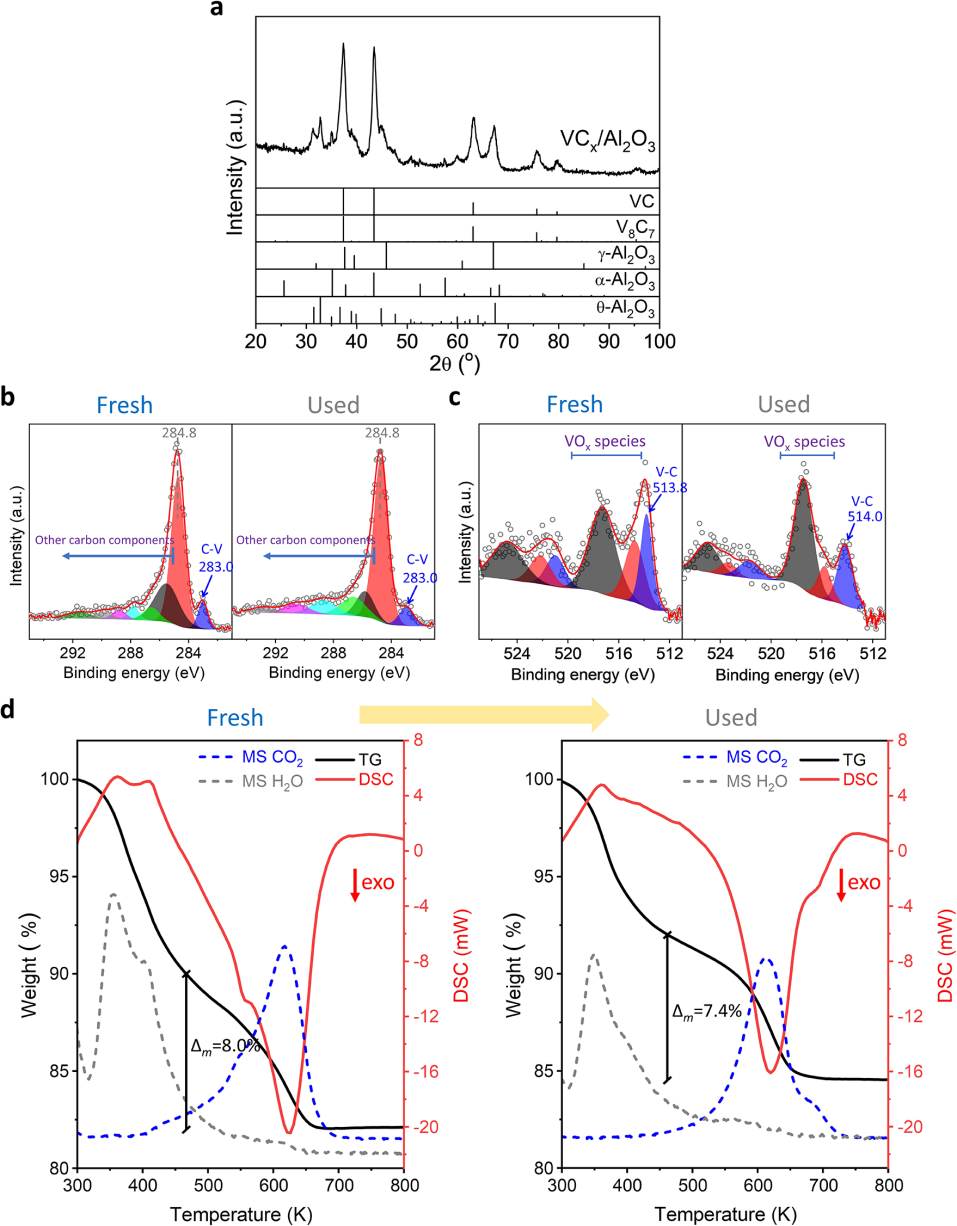
Characterization of used VC_*x*_/Al_2_O_3_ catalyst after RWGS reaction: (a) XRD, (b) C *1s* XPS level, (c) V *2p* XPS level, and (d) TGA-MS analysis under air of fresh and used VC_*x*_/Al_2_O_3_. XPS of fresh VC_*x*_/Al_2_O_3_ was plotted for comparison. Reaction conditions: *T* = 523–873 K, CO_2_/H_2_/N_2_ = 1/3/1; GHSV = 3000 h^–1^; *P* = 0.1 MPa; m = 300 mg.

Finally, a RWGS stability test was performed using the most efficient catalyst, VC_*x*_/Al_2_O_3_, at 873 K for 100 h. Constant values of approximately 51% CO_2_ conversion and 99.96% CO selectivity were found (Figure 3f). Moreover, in order to determine the possible coke formation during the RWGS, TGA-MS analysis of fresh and used VC_*x*_/Al_2_O_3_ was conducted (Figure 4d). The TGA-MS profiles of the fresh and used catalysts were similar. Initially, the loss of H_2_O was noted. Then, CO_2_ formation took place with maxima at about 623 K, likely related to the combustion of residual carbonaceous deposits formed during the catalyst preparation. This indicates that after the long RWGS stability test, no additional carbon deposition occurred on the VC_*x*_/Al_2_O_3_ catalyst.

### 3.3 MSR Reaction

All supported catalysts exhibited higher methanol conversion than bulk VC_*x*_ under the reaction conditions employed (Figure 5a,b). In all cases, the conversion was low at 573 K and increased with temperature, with the highest value observed for VC_*x*_/ZrO_2_ (Figure 5b). Across the temperature range studied, CH_4_ was always the main product evolved (Figures 5b,c and S11), as previously observed for bulk VC_*x*_,^22^ which could be produced via the methanol decomposition reaction outlined as follows

6 $$CH_{3}OH_{\left(\mathrm{ad}\right)}\to CH_{4\left(g\right)}+O_{\left(\mathrm{ad}\right)}$$

**Figure 5:**
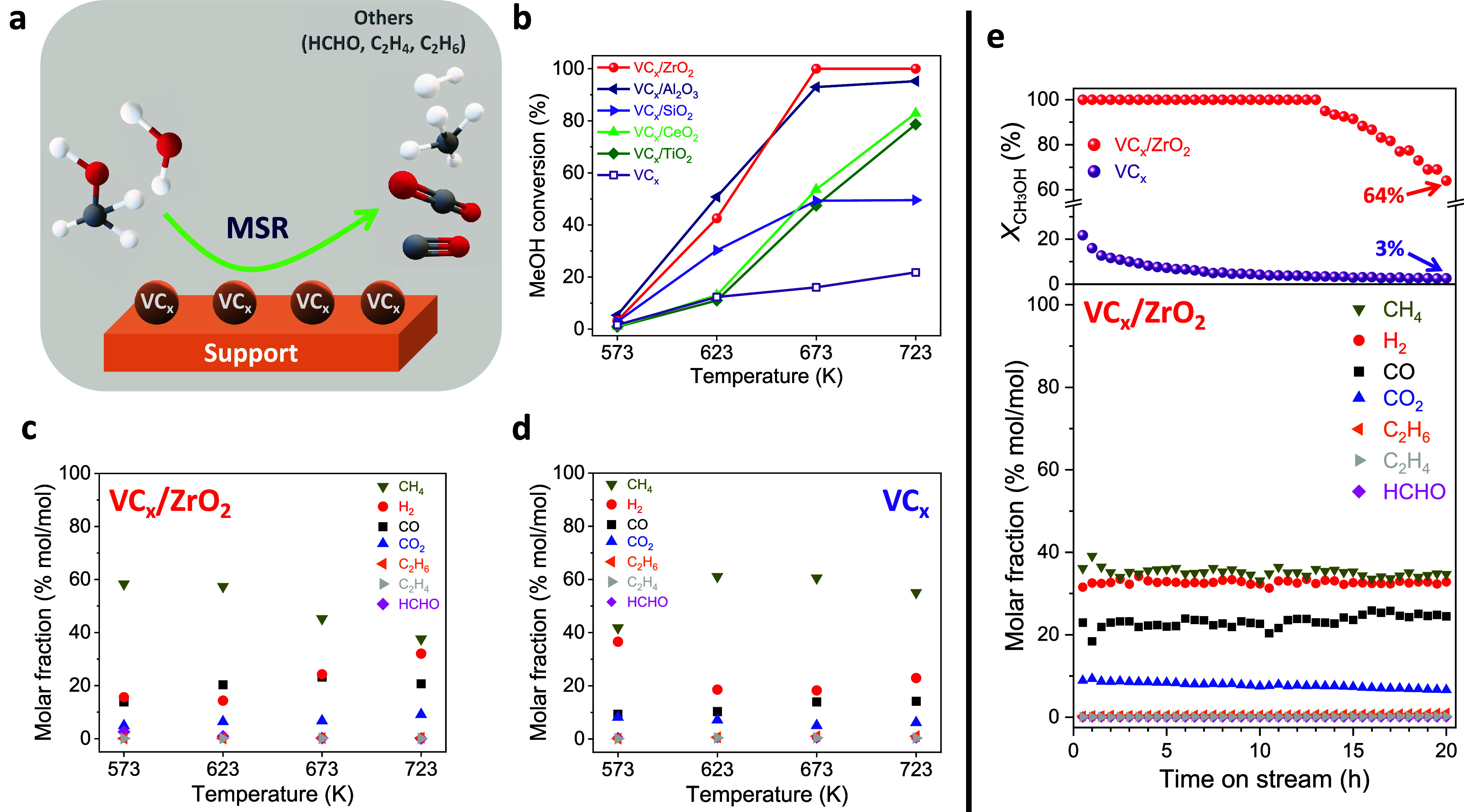
Catalytic behavior of supported VC_*x*_ catalysts in the MSR reaction: (a) Schematic of MSR reaction, (b) CH_3_OH conversion, (c) product molar fraction over VC_*x*_/ZrO_2_, (d) product molar fraction over bulk VC_*x*_, and (e) stability test over VC_*x*_/ZrO_2_ at 723 K for 20 h. Reaction conditions: CH_3_OH/H_2_O/N_2_ = 1/1/1.2; GHSV = 2500 h^–1^; *P* = 0.1 MPa; *m* = 300 mg.

Subsequently, the adsorbed O* species generated during methanol decomposition may react with H_2_, regenerating the original VC_*x*_ surface. These results indicate that VC_*x*_ promotes the decomposition of CH_3_OH through C–O bond cleavage of intermediate methoxy species, producing CH_4_ and surface oxygen.^26^

Among supported catalysts, VC_*x*_/ZrO_2_ exhibited the highest H_2_ yield (Figures 5c and S11). For this sample, a slight decrease in CH_4_ formation and a corresponding increase in H_2_ formation was observed with increasing temperature (Figure 5c). This behavior can be attributed to the ability of ZrO_2_ support to stabilize surface methoxy groups, thereby promoting H_2_ formation and suppressing methanation, as previously observed for Mo_2_C/ZrO_2_ catalysts during MSR.^54^ Differences in product distribution likely depend on the nature of the support, as properties such as acidity and the presence of defect sites can play a significant role in the reaction mechanism.

In addition to CH_4_ and H_2_, CO, CO_2_ and HCHO were found in variable amounts depending on the catalyst used. Moreover, very small amounts of C_2_H_4_ and C_2_H_6_ were also observed (Figures 5c and S11). The dehydrogenation of methanol (eq 7) leads to HCHO formation, which can decompose producing CO and H_2_ (eq 8).

7 $$CH_{3}OH_{\left(g\right)}\to \mathrm{HCH}O_{\left(g\right)}+H_{2\left(g\right)}$$

8 $$\mathrm{HCH}O_{\left(g\right)}\to CO_{\left(g\right)}+H_{2\left(g\right)}$$

On the other hand, once CO is formed, it could alternatively react with H_2_O producing CO_2_ and H_2_. This reaction, as well as the HCHO decomposition, would be favored by increasing the temperature. Furthermore, at low temperatures, the product distribution could be influenced by the Water Gas Shift (WGS) reaction equilibrium (eq 9).

$$CO_{\left(g\right)}+H_{2}O_{\left(g\right)}⇄CO_{2\left(g\right)}+H_{2\left(g\right)}$$ 9

Although VC_*x*_/ZrO_2_ was the most active catalyst for MSR among those tested in this work, and even outperformed many catalysts reported in the literature (achieving 100% methanol conversion at 673 K), is less suitable for producing H_2_ and CO/CO_2_-rich products, which are the desired products in conventional reforming processes typically catalyzed by Cu-based catalysts (e.g., Cu/ZnO/Al_2_O_3_, see Table S2). However, its high CH_4_/H_2_ yield ratio suggest potential in alternative applications, such as converting stored chemical energy (methanol) back into methane, which can subsequently be utilized within existing natural gas infrastructure.

To assess the stability of the samples, the reaction time was extended to 20 h at 723 K (Figures 5e and S12). All catalysts displayed severe deactivation. This behavior was previously observed for bulk VC_*x*_ *-* and Mo_2_C-based catalysts under MSR conditions.^22,55,56^ The evolution of the product distribution over time is also presented for all catalysts in Figures 5e and S12. As expected, in most cases, the decrease in the methanol conversion was accompanied by a significant variation in the product distribution. However, VC_*x*_/ZrO_2_, which showed the highest H_2_ yield and a methanol conversion of 64% after 20 h at 723 K, showed almost no variation in CH_4_ and H_2_ concentrations over time (Figure 5e). For a further analysis of carbon deposits formed on VC_*x*_/ZrO_2_ during the MSR reaction, TGA-MS experiments were performed before and after the MSR catalytic test (Figure 6a); after the initial loss of H_2_O, CO_2_ evolution was observed in both cases. A larger amount of CO_2_ was formed in the used catalyst at slightly higher temperature (623–650 K) than for the fresh sample (573–623 K). This indicates the formation of carbon deposits on VC_*x*_/ZrO_2_ during the MSR reaction. Moreover, the simultaneous evolution of water at this high temperature points to the presence of C_*x*_H_*y*_ species.

**Figure 6:**
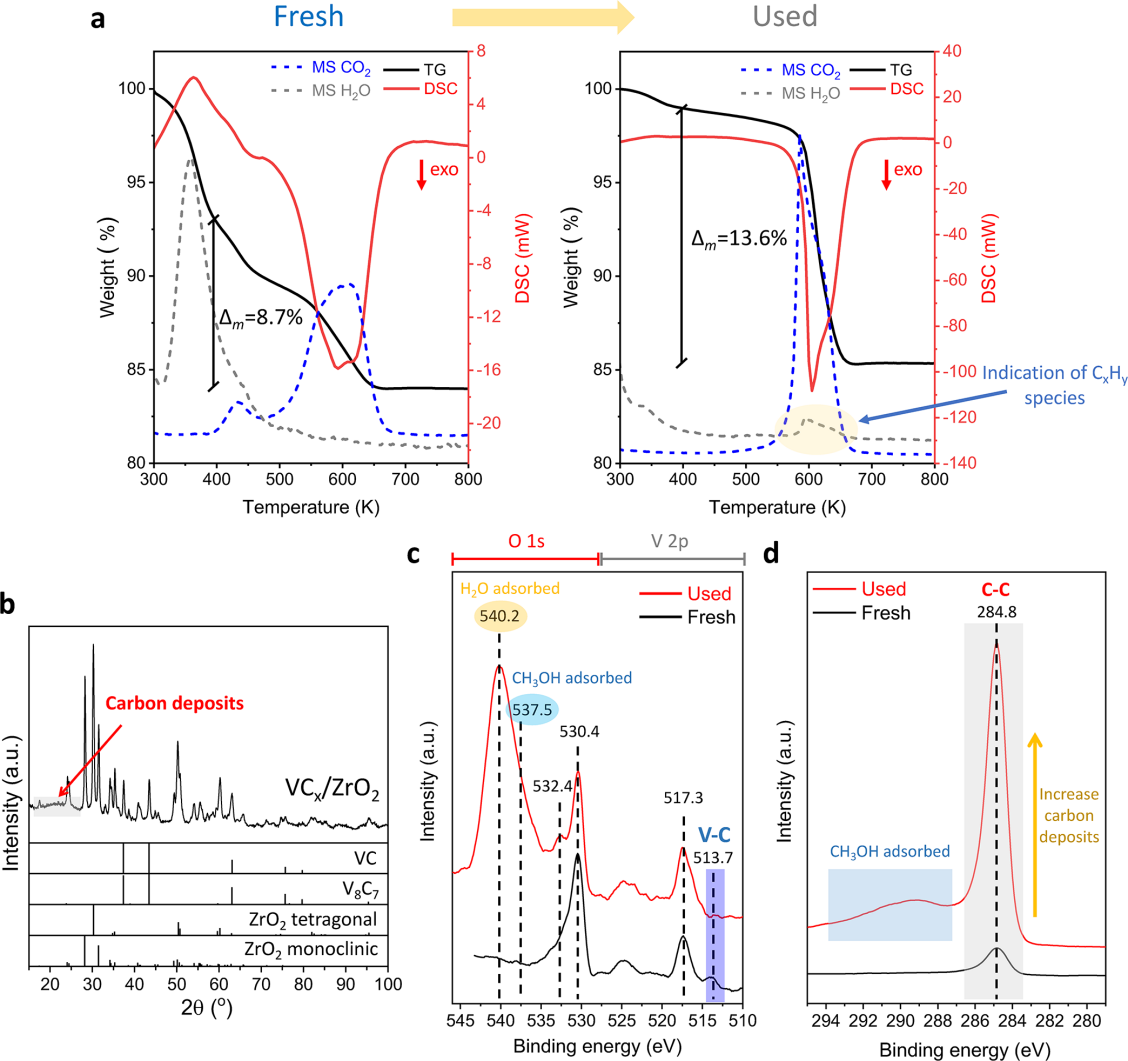
Characterization of used VC_*x*_/ZrO_2_ catalyst after MSR reaction: (a) TGA-MS analysis under air of fresh and used VC_*x*_/ZrO_2_, (b) XRD pattern, (c) V *2p*—O *1s* XPS levels (combined) and (d) C *1s* XPS level. XPS of fresh catalyst was plotted for comparison. Reaction conditions: *T* = 573–723 K, CH_3_OH/H_2_O/N_2_ = 1/1/1.2; GHSV = 2500 h^–1^; *P* = 0.1 MPa; *m* = 300 mg.

The characterization of catalysts by XRD after the MSR (Figures 6b and S13), pointed out that in all cases the VC_*x*_ crystallite size on the used and the fresh catalyst was similar. Additionally, no XRD peaks corresponding to crystalline VO_*x*_ species were detected in any case. However, XRD patterns of VC_*x*_/ZrO_2_ and VC_*x*_/TiO_2_ indicated some structural changes in the supports after the MSR reaction (Figures 6b and S13d). For VC_*x*_/ZrO_2_, more intense peaks corresponding to monoclinic ZrO_2_ were observed (Figure 6b). For VC_*x*_/TiO_2_, small peaks corresponding to rutile and anatase TiO_2_ were detected after the MSR reaction, likely resulting from a partial transformation of the initial solid solution, (Ti_0.9_V_0.1_)_2_O_3_, present in the fresh VC_*x*_/TiO_2_ (Figure S13d). Furthermore, the presence of carbonaceous deposits can be inferred from the appearance of a broad peak at 2θ = 25.0° in all cases, which is consistent with the results obtained for bulk group 5 TMC catalysts.^22^ CH_4_ decomposition likely contributed to coke formation. Additionally, a significant reduction in the surface area (*S*_BET_) values of used catalysts was observed when compared to fresh catalysts (Table 1), which could be related (at least in part) to the coke deposition during the MSR catalytic test.

Figures 6c and S14 display the XPS profiles of the V *2p* and O *1s* core levels for the catalysts after the MSR reaction. The intensity of the V *2p*_*3/2*_ band at 513.5–513.8 eV, associated with the presence of VC_*x*_ species, decreased compared to the fresh catalysts, while the signal at 517.1–517.5 eV, attributed to oxy-vanadium species, increased. Additionally, new bands appearing at 537–542 eV (O *1s*) were attributed to adsorbed CH_3_OH and H_2_O species.^57^ The C *1s* core level spectra of the used catalysts (Figures 6d and S15) showed that the peak corresponding to carbidic species (C *1s* at ∼ 283.0 eV) is difficult to be observed, likely due to overlap with the main C *1s* peak at 284.8 eV, whose intensity increased significantly relative to the fresh samples. This increase can be attributed to the formation of carbon deposits during the MSR catalytic test, as previously discussed.

### 3.4 DFT Calculations to Understand Coke Formation

To elucidate the experimental observations of coke formation, DFT calculations were performed to investigate the decomposition mechanisms of CO_2_ and CH_3_OH on VC and V_8_C_7_ slab models. Initially, we identified all plausible pathways for atomic carbon formation on V_8_C_7_ from CO_2_ and CH_3_OH. For CO_2_ decomposition, only the direct C–O dissociation pathway (CO_2_ → CO → C) was considered. In contrast, CH_3_OH decomposition may proceed via multiple pathways, depending on which bond is cleaved initially. We explored several bond-breaking elementary steps in methanol decomposition, calculated the corresponding energy barriers, and mapped the lowest energy pathway (Figure S16).

Our results indicated that the most favorable route for C* formation from CH_3_OH on both VC and V_8_C_7_ begins with the breaking of the O–H bond, forming CH_3_O* (Figure 7). On VC, this pathway continues with C–H bond cleavage to form CH_2_O* and subsequently CHO*, followed by C–O bond scission to produce CH*, and ultimately C*. In contrast, on V_8_C_7_, the CH_3_O* species preferentially undergoes C–O bond scission prior to C–H activation, yielding CH_3_* first, which is subsequently dehydrogenated to form C*. Notably, on the V_8_C_7_ model, the vacancy site is not always available for subsequent bond dissociation steps. Specifically, after CO_2_* dissociation, the vacancy is occupied by an O* atom, so the next C–O bond scission occurs adjacent to the vacancy. Similarly, for CH_3_OH, the vacancy site facilitates three of the five bond dissociation steps, while the remaining two occur at sites adjacent to the vacancy. The transition state geometries of the most favorable reaction steps are presented in Figure S17.

**Figure 7:**
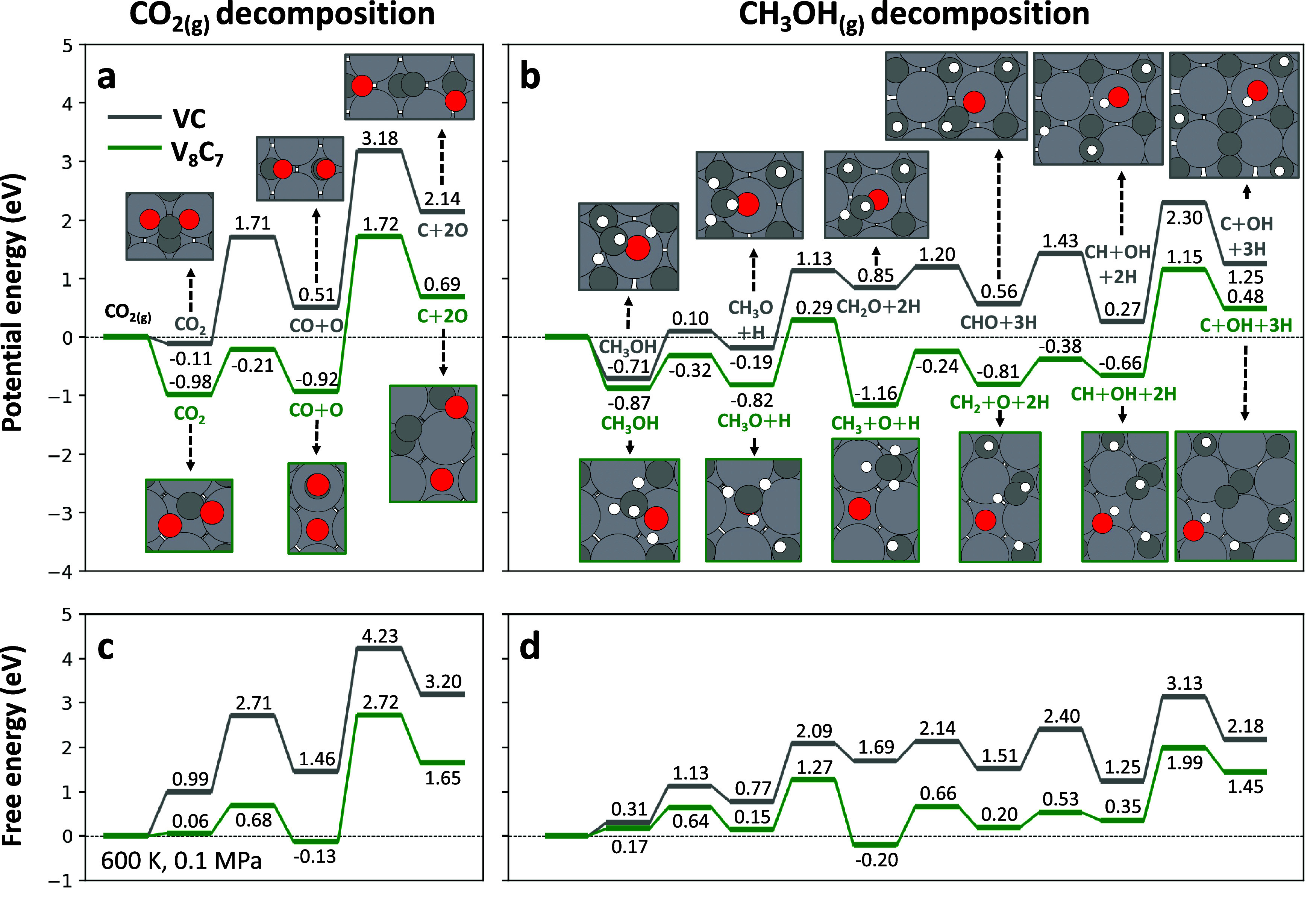
(a, b) Potential energy diagrams and (c, d) free energy diagrams for (a, c) CO_2_ decomposition and (b, d) CH_3_OH decomposition. Gray and green lines correspond to VC and V_8_C_7_, respectively. The free energy diagrams have been computed at 600 K and 0.1 MPa and the values reported are in units of eV. The reference states used for the potential and free energy diagrams are the gas-phase reactant species.

Comparing the potential and Gibbs free energy profiles for CO_2_ and CH_3_OH decomposition reveals that methanol decomposition is significantly more favorable than CO_2_ decomposition on both VC and V_8_C_7_ (Figure 7). This finding aligns with our experimental observations, including stability tests, XRD, XPS, and TGA-MS profiles. Specifically, C–O bond cleavage in adsorbed CO* during CO_2_ decomposition has high potential energy barriers of 2.67 eV on VC and 2.64 eV on V_8_C_7_ (Figure 7a), making it unlikely to occur under the reaction conditions tested (Figure 3). In contrast, when carbon forms bonds with hydrogen atoms, as in CH_3_O*, the C–O bond becomes significantly weaker. For example, the C–O bond dissociation of CH_3_O* on V_8_C_7_ has a potential energy barrier of only 1.11 eV (Figure 7b), which is accessible under reaction conditions (Figure 5).

The presence of carbon vacancies systematically reduces the energy barriers for both CO_2_ and CH_3_OH decompositions and stabilizes C* and CH* species. Specifically, the overall potential energy barrier for C* formation from CO_2_ decreases from 3.18 eV on VC to 1.72 eV on V_8_C_7_, and for C* formation from CH_3_OH, it decreases from 2.30 eV on VC to 1.15 eV on V_8_C_7_. Therefore, coke formation is expected to be more pronounced on VC_*x*_ samples with a higher proportion of the V_8_C_7_ phase.

Furthermore, the energy profiles suggest that coke deposits under MSR conditions likely consist of C_*x*_H_y_* species rather than solely C* atoms, as the CH* → C* dissociation requires overcoming high potential energy barriers of 2.03 eV on VC and 1.81 eV on V_8_C_7_. This is supported by the TGA-MS profile of the used VC_*x*_/ZrO_2_ catalyst in MSR, which showed the presence of H_2_O at the CO_2_ release temperature (Figure 6a), indicating that the formed coke may comprise C_*x*_H_y_* species. The CH* intermediate could initiate the formation of long, partially hydrogenated C_*x*_H_*y*_ rings or chains, which can deactivate the VC_*x*_ catalyst. Overall, the DFT calculations demonstrated that VC_*x*_ catalysts are more prone to coking during CH_3_OH decomposition than during CO_2_ decomposition.

## 4 Conclusions

In this study, a series of vanadium carbide catalysts supported on Al_2_O_3_, SiO_2_, TiO_2_, CeO_2_ and ZrO_2_ were successfully synthesized and evaluated for CO_2_ and CH_3_OH conversion under the RWGS and MSR conditions, respectively.

Under RWGS conditions, all catalysts exhibited high selectivity for CO, reaching up to 100% at 873 K. Compared to bulk VC_*x*_, the supported catalysts demonstrated significantly higher CO production per mol of V. This enhanced activity is attributed to their smaller VC_*x*_ particle sizes and consequently higher concentrations of carbon vacancies, which facilitate the adsorption and dissociation of CO_2_ and H_2_,^19^ as well as to favorable support-VC_*x*_ interfacial effects. Among the studied samples, VC_*x*_/Al_2_O_3_ showed the best performance, exhibiting high stability and no evidence of carbon deposition after 100 h on stream at 873 K. The superior behavior of this catalyst could be attributed to the −OH groups on the Al_2_O_3_ surface, which likely assist CO_2_ activation via bicarbonate and formate intermediates at the carbide-support interface.^52^

In the MSR reaction, the supported VC_*x*_ catalysts achieved higher methanol conversions than bulk VC_*x*_, with VC_*x*_/ZrO_2_ being the most active in the 673–723 K range. However, in all cases, CH_4_ was the main product, and the catalysts suffered substantial deactivation due to coke formation.

Postreaction characterization confirmed the presence of VC_*x*_ phases in all spent catalysts via XRD, with no detectable crystalline VO_*x*_ species. XPS analysis revealed that surface VC_*x*_ was preserved under RWGS conditions but underwent partial oxidation during MSR.

DFT calculations offered mechanistic insights into coke formation pathways on VC and V_8_C_7_ surfaces. The computed energy landscapes revealed that methanol decomposition proceeds via pathways with significantly lower energy barriers than CO_2_ decomposition, and thus CH_3_OH is a more potent precursor for coke. The presence of carbon vacancies was found to further lower the energy barriers, thereby increasing the likelihood of coke formation on V_8_C_7_-rich VC_*x*_ samples. Additionally, the results suggest that coke species formed under MSR conditions are partially hydrogenated C_*x*_H_y_* intermediates rather than pure C* atoms.

Overall, this work demonstrates that supported VC_*x*_ catalysts are highly active and selective for the RWGS, while bulk VC_*x*_ exhibits lower activity. Conversely, under MSR conditions, VC_*x*_ catalysts are more susceptible to coking, particularly when V_8_C_7_ phases dominate. The higher catalytic activity of the supported materials arises from the combined effects of smaller crystallite size (and hence more carbon vacancies) and specific metal–support interfacial interactions that enhance CO_2_ and CH_3_OH activation. These findings highlight the importance of tailoring the catalyst surface structure and metal–support interface to minimize coke formation and improve the long-term catalytic stability of transition-metal carbide catalysts.

## Supplementary Material



## Notes

This includes additional details on the DFT slab models, N_2_ adsorption–desorption isotherms, pore size distribution, Raman spectra, TEM characterization, XPS profiles, XRD patterns, product distribution, catalytic tests, methanol and CO_2_ decomposition pathways and transition state configurations (PDF)

## References

[1] Hwu H. H., Chen J. G. (2005). Surface Chemistry
of Transition Metal Carbides. Chem. Rev..

[2] Levy R. B., Boudart M. (1973). Platinum-Like Behavior
of Tungsten Carbide in Surface
Catalysis. Science.

[3] Prats H., Piñero J. J., Viñes F., Bromley S. T., Sayós R., Illas F. (2019). Assessing the Usefulness of Transition Metal Carbides for Hydrogenation
Reactions. Chem. Commun..

[4] Kunkel C., Vines F., Ramírez P. J., Rodriguez J. A., Illas F. (2019). Combining Theory and Experiment for
Multitechnique Characterization
of Activated CO_2_ on Transition Metal Carbide (001) Surfaces. J. Phys. Chem. C.

[5] Quesne M. G., Roldan A., De Leeuw N. H., Catlow C. R. A. (2018). Bulk and Surface
Properties of Metal Carbides: Implications for Catalysis. Phys. Chem. Chem. Phys..

[6] Prats H., Stamatakis M. (2023). Breaking Linear
Scaling Relationships with Transition
Metal Carbides. Catal. Sci. Technol..

[7] Li Z., Choi J. S., Wang H., Lepore A. W., Connatser R. M., Lewis S. A., Meyer H. M., Santosa D. M., Zacher A. H. (2017). Sulfur-Tolerant
Molybdenum Carbide Catalysts Enabling Low-Temperature Stabilization
of Fast Pyrolysis Bio-Oil. Energy Fuels.

[8] Claridge J. B., York A. P. E., Brungs A. J., Marquez-Alvarez C., Sloan J., Tsang S. C., Green M. L. H. (1998). New Catalysts
for the Conversion of Methane to Synthesis Gas: Molybdenum and Tungsten
Carbide. J. Catal..

[9] Führer M., Van Haasterecht T., Bitter J. H. (2020). Molybdenum and Tungsten Carbides
Can Shine Too. Catal. Sci. Technol..

[10] Lin Z., Denny S. R., Chen J. G. (2021). Transition
Metal Carbides and Nitrides
as Catalysts for Thermochemical Reactions. J.
Catal..

[11] Pang J., Sun J., Zheng M., Li H., Wang Y., Zhang T. (2019). Transition
Metal Carbide Catalysts for Biomass Conversion: A Review. Appl. Catal., B.

[12] Chen J. G., Frühberger B., Weisel M. D., Baumgartner J. E., De Vries B. D. (1996). Characterization of the Electronic and Catalytic Properties
of the Vanadium Carbide: A Comparative Study of VC/V(110) Model Surfaces
and VC Powder Materials. Chem. Transition Metal
Carbides Nitrides.

[13] Choi J. G. (1999). Ammonia
Decomposition over Vanadium Carbide Catalysts. J. Catal..

[14] Huang T., Yu J., Han J., Zhang Z., Xing Y., Wen C., Wu X., Zhang Y. (2015). Oxygen Reduction Catalytic Characteristics of Vanadium
Carbide and Nitrogen Doped Vanadium Carbide. J. Power Sources.

[15] Yu J., Gao X., Chen G., Yuan X. (2016). Electrocatalytic Performance of Commercial
Vanadium Carbide for Oxygen Reduction Reaction. Int. J. Hydrogen Energy.

[16] Tian L., Min S., Wang F., Zhang Z. (2020). Enhanced Photocatalytic Hydrogen
Evolution on TiO_2_ Employing Vanadium Carbide as an Efficient
and Stable Cocatalyst. Int. J. Hydrogen Energy.

[17] Peng X., Hu L., Wang L., Zhang X., Fu J., Huo K., Lee L. Y. S., Wong K. Y., Chu P. K. (2016). Vanadium Carbide
Nanoparticles Encapsulated in Graphitic Carbon Network Nanosheets:
A High-Efficiency Electrocatalyst for Hydrogen Evolution Reaction. Nano Energy.

[18] Kwon H., Thompson L. T., Eng J., Chen J. G. (2000). N-Butane
Dehydrogenation
over Vanadium Carbides: Correlating Catalytic and Electronic Properties. J. Catal..

[19] Pajares A., Prats H., Romero A., Viñes F., de la Piscina P. R., Sayós R., Homs N., Illas F. (2020). Critical Effect
of Carbon Vacancies on the Reverse Water Gas Shift Reaction over Vanadium
Carbide Catalysts. Appl. Catal., B.

[20] Prats H., Pajares A., Viñes F., Ramírez de la Piscina P., Sayós R., Homs N., Illas F. (2024). On the Capabilities
of Transition Metal Carbides for Carbon Capture and Utilization Technologies. ACS Appl. Mater. Interfaces.

[21] Pajares A., Ramírez de la Piscina P., Homs N. (2024). Selective Reduction
of CO_2_ to CO over Alumina-Supported Catalysts of Group
5 Transition Metal Carbides. Appl. Catal. A-Gen..

[22] Pajares A., Ramírez de la Piscina P., Homs N. (2024). Catalytic Behaviour
of Transition Metal Carbides of Group 5 in the Methanol Steam Reforming. Int. J. Hydrogen Energy.

[23] Hu B., Shu R., Tian Z., Wang C., Chen Y., Xu Y. (2024). Enhancement
of Hydrogen Production via Methanol Steam Reforming Using a Ni-Based
Catalyst Supported by Spongy Mesoporous Alumina. Green Chem..

[24] Shu R., Xie L., Hu B., Tian Z., Wang C., Chen Y., Xu Y. (2024). Reinforcement of Methanol Catalytic Reforming for Hydrogen Production
through Ru-Based Carbon-Coated CeO_2_ Catalyst. Fuel.

[25] Xie L., Hu B., Shu R., Tian Z., Chen Y., Wang C. (2023). Effect of
Oxygen Vacancy Influenced by CeO_2_ Morphology on the Methanol
Catalytic Reforming for Hydrogen Production. Int. J. Hydrogen Energy.

[26] Zellner M. B., Hwu H. H., Chen J. G. (2005). Comparative
Studies of Methanol Decomposition
on Carbide-Modified V(110) and Ti(0001). Surf.
Sci..

[27] Perdew J. P., Burke K., Ernzerhof M. (1996). Generalized
Gradient Approximation
Made Simple. Phys. Rev. Lett..

[28] Grimme S., Antony J., Ehrlich S., Krieg H. (2010). A Consistent and Accurate
Ab Initio Parametrization of Density Functional Dispersion Correction
(DFT-D) for the 94 Elements H-Pu. J. Chem. Phys..

[29] Blöchl P. E. (1994). Projector
Augmented-Wave Method. Phys. Rev. B.

[30] Kresse G., Joubert D. (1999). From Ultrasoft Pseudopotentials to the Projector Augmented-Wave
Method. Phys. Rev. B.

[31] Chong X., Jiang Y., Zhou R., Feng J. (2016). The Effects of Ordered
Carbon Vacancies on Stability and Thermo-Mechanical Properties of
V_8_C_7_ Compared with VC. Sci. Rep..

[32] Garrido
Torres J. A., Jennings P. C., Hansen M. H., Boes J. R., Bligaard T. (2019). Low-Scaling Algorithm for Nudged Elastic Band Calculations
Using a Surrogate Machine Learning Model. Phys.
Rev. Lett..

[33] Hjorth
Larsen A., JØrgen Mortensen J., Blomqvist J., Castelli I. E., Christensen R., Dułak M., Friis J., Groves M. N., Hammer B., Hargus C., Hermes E. D., Jennings P. C., Bjerre Jensen P., Kermode J., Kitchin J. R., Leonhard Kolsbjerg E., Kubal J., Kaasbjerg K., Lysgaard S., Bergmann
Maronsson J., Maxson T., Olsen T., Pastewka L., Peterson A., Rostgaard C., SchiØtz J., Schütt O., Strange M., Thygesen K. S., Vegge T., Vilhelmsen L., Walter M., Zeng Z., Jacobsen K. W. (2017). The Atomic
Simulation Environment—a Python Library for Working with Atoms. J. Phys.-Condens. Matter.

[34] Eklund P., Sridharan M., Singh G., Bøttiger J. (2009). Thermal Stability
and Phase Transformations of γ-/Amorphous-Al_2_O_3_ Thin Films. Plasma Process. Polym..

[35] Boullosa-Eiras S., Vanhaecke E., Zhao T., Chen D., Holmen A. (2011). Raman Spectroscopy
and X-Ray Diffraction Study of the Phase Transformation of ZrO_2_–Al_2_O_3_ and CeO_2_–Al_2_O_3_ Nanocomposites. Catal.
Today.

[36] Fu Q., Sarapulova A., Trouillet V., Zhu L., Fauth F., Mangold S., Welter E., Indris S., Knapp M., Dsoke S., Bramnik N., Ehrenberg H. (2019). In Operando
Synchrotron Diffraction and in Operando X-Ray Absorption Spectroscopy
Investigations of Orthorhombic V_2_O_5_ Nanowires
as Cathode Materials for Mg-Ion Batteries. J.
Am. Chem. Soc..

[37] Liu G., Zhao Z. J., Wu T., Zeng L., Gong J. (2016). Nature of
the Active Sites of VO_x_/Al_2_O_3_ Catalysts
for Propane Dehydrogenation. ACS Catal..

[38] Liu Q. Q., Fan C. Y., Tang H., Ma T. D., Shen J. Y. (2016). One-Step
Synthesis of Recycled 3D CeVO_4_/RGO Composite Aerogels for
Efficient Degradation of Organic Dyes. RSC Adv..

[39] Martínez-Huerta M. V., Deo G., Fierro J. L. G., Bañares M. A. (2008). Operando Raman-GC Study on the Structure–Activity
Relationships in V_5+_/CeO_2_ Catalyst for Ethane
Oxidative Dehydrogenation: The Formation of CeVO_4_. J. Phys. Chem. C.

[40] Schilling C., Hofmann A., Hess C., Ganduglia-Pirovano M. V. (2017). Raman Spectra
of Polycrystalline CeO_2_: A Density Functional Theory Study. J. Phys. Chem. C.

[41] Schipporeit S., Mergel D. (2018). Spectral Decomposition
of Raman Spectra of Mixed-Phase
TiO2 Thin Films on Si and Silicate Substrates. J. Raman Spectrosc..

[42] Meunier F., Delporte P., Heinrich B., Bouchy C., Crouzet C., Pham-Huu C., Panissod P., Lerou J. J., Mills P. L., Ledoux M. J. (1997). Synthesis and Characterization
of High Specific Surface
Area Vanadium Carbide; Application to Catalytic Oxidation. J. Catal..

[43] Kanervo J. M., Harlin M. E., Krause A. O. I., Bañares M. A. (2003). Characterisation
of Alumina-Supported Vanadium Oxide Catalysts by Kinetic Analysis
of H_2_-TPR Data. Catal. Today.

[44] Harlin M. E., Niemi V. M., Krause A. O. I. (2000). Alumina-Supported
Vanadium Oxide
in the Dehydrogenation of Butanes. J. Catal..

[45] Huang X., Zhang G., Dong F., Tang Z. (2018). The Remarkable Promotional
Effect of Sn on CeVO_4_ Catalyst for Wide Temperature NH_3_-SCR Process by Citric Acid-Assisted Solvothermal Synthesis
and Post-Hydrothermal Treatment. Catal. Sci.
Technol..

[46] Arena F., Frusteri F., Parmaliana A. (1999). Structure and Dispersion of Supported-Vanadia
Catalysts. Influence of the Oxide Carrier. Appl.
Catal. A-Gen..

[47] Sonia F. J., Kalita H., Aslam M., Mukhopadhyay A. (2017). Correlations
between Preparation Methods, Structural Features and Electrochemical
Li-Storage Behavior of Reduced Graphene Oxide. Nanoscale.

[48] Enterría M., Martín-Jimeno F. J., Suárez-García F., Paredes J. I., Pereira M. F. R., Martins J. I., Martínez-Alonso A., Tascón J. M. D., Figueiredo J. L. (2016). Effect of Nanostructure on the Supercapacitor
Performance of Activated Carbon Xerogels Obtained from Hydrothermally
Carbonized Glucose-Graphene Oxide Hybrids. Carbon.

[49] Zhu Y., Murali S., Stoller M. D., Ganesh K. J., Cai W., Ferreira P. J., Pirkle A., Wallace R. M., Cychosz K. A., Thommes M., Su D., Stach E. A., Ruoff R. S. (2011). Carbon-Based
Supercapacitors Produced by Activation of Graphene. Science.

[50] Figueiredo J. L., Pereira M. F. R., Freitas M. M. A., Órfão J. J. M. (1999). Modification
of the Surface Chemistry of Activated Carbons. Carbon.

[51] Leclercq L., Almazouari A., Dufour M., Leclercq G. (1996). Carbide-Oxide
Interactions
in Bulk and Supported Tungsten Carbide Catalysts for Alcohol Synthesis. Chem. Transit. Metal Carbides Nitrides.

[52] Pajares A., Andrade-Arvizu J., Jain D., Monai M., Lefevere J., de la Piscina P. R., Homs N., Michielsen B. (2024). Exploring
the 3D Printing of Molybdenum Carbide-Based Catalysts for the Reverse
Water Gas Shift Reaction: A Multi Scale Study. Chem. Eng. J..

[53] Juneau M., Pope C., Liu R., Porosoff M. D. (2021). Support
Acidity
as a Descriptor for Reverse Water-Gas Shift over Mo_2_C-Based
Catalysts. Appl. Catal. A-Gen..

[54] Lin S. S. Y., Thomson W. J., Hagensen T. J., Ha S. Y. (2007). Steam Reforming
of Methanol Using Supported Mo_2_C Catalysts. Appl. Catal. A-Gen..

[55] Ma Y., Guan G., Shi C., Zhu A., Hao X., Wang Z., Kusakabe K., Abudula A. (2014). Low-Temperature Steam
Reforming of Methanol to Produce Hydrogen over Various Metal-Doped
Molybdenum Carbide Catalysts. Int. J. Hydrogen
Energy.

[56] Cao J., Ma Y., Guan G., Hao X., Ma X., Wang Z., Kusakabe K., Abudula A. (2016). Reaction Intermediate
Species during
the Steam Reforming of Methanol over Metal Modified Molybdenum Carbide
Catalysts. Appl. Catal., B.

[57] Pellegrin E., Perez-Dieste V., Escudero C., Rejmak P., Gonzalez N., Fontsere A., Prat J., Fraxedas J., Ferrer S. (2020). Water/Methanol
Solutions Characterized by Liquid μ-Jet XPS and DFT—The
Methanol Hydration Case. J. Mol. Liq..

