# Supplementary material for: Supported Vanadium Carbide Catalysts for Reverse Water Gas Shift and Methanol Steam Reforming: Activity, Stability, and Coking Pathways
Source: ACS Appl Mater Interfaces. 2025 Nov 21;17(49):66595–607. doi: 10.1021/acsami.5c16601 (PMC12874385; doi:10.1021/acsami.5c16601)
Supplement: Supplementary file 1 [file am5c16601_si_001.pdf]

Supported Vanadium Carbide Catalysts for Reverse Water Gas Shift and Methanol Steam Reforming: Activity, Stability, and Coking Pathways

Arturo Pajares[1,*], Sai Sharath Yadavalli[2], Hector Prats[2,3*], Pilar Ramírez de la Piscina[4], Michail Stamatakis[2], Narcís Homs[4,5]

[1]*Materials & Chemistry, Flemish Institute for Technological Research (VITO NV), Boeretang 200, 2400 Mol, Belgium.*

[2]*Department of Chemistry, Inorganic Chemistry Laboratory, University of Oxford, South Parks Road, Oxford OX1 3QZ, UK.*

[3]*Institute of Materials Chemistry, Technische Universität Wien, Getreidemarkt 9/165, 1060 Vienna, Austria*

[4]*Departament de Química Inorgànica i Orgànica, secció de Química Inorgànica & Institut de Nanociència i Nanotecnología (IN2UB), Universitat de Barcelona, Martí i Franquès 1-11, 08028 Barcelona, Spain.*

[5]*Catalonia Institute for Energy Research (IREC), Jardins de les Dones de Negre 1, 08930 Barcelona, Spain.*

**Corresponding authors: Arturo Pajares (arturo.pajares@vito.be) and Hector Prats (hector.prats@tuwien.ac.at)*

1. Supplementary Figures

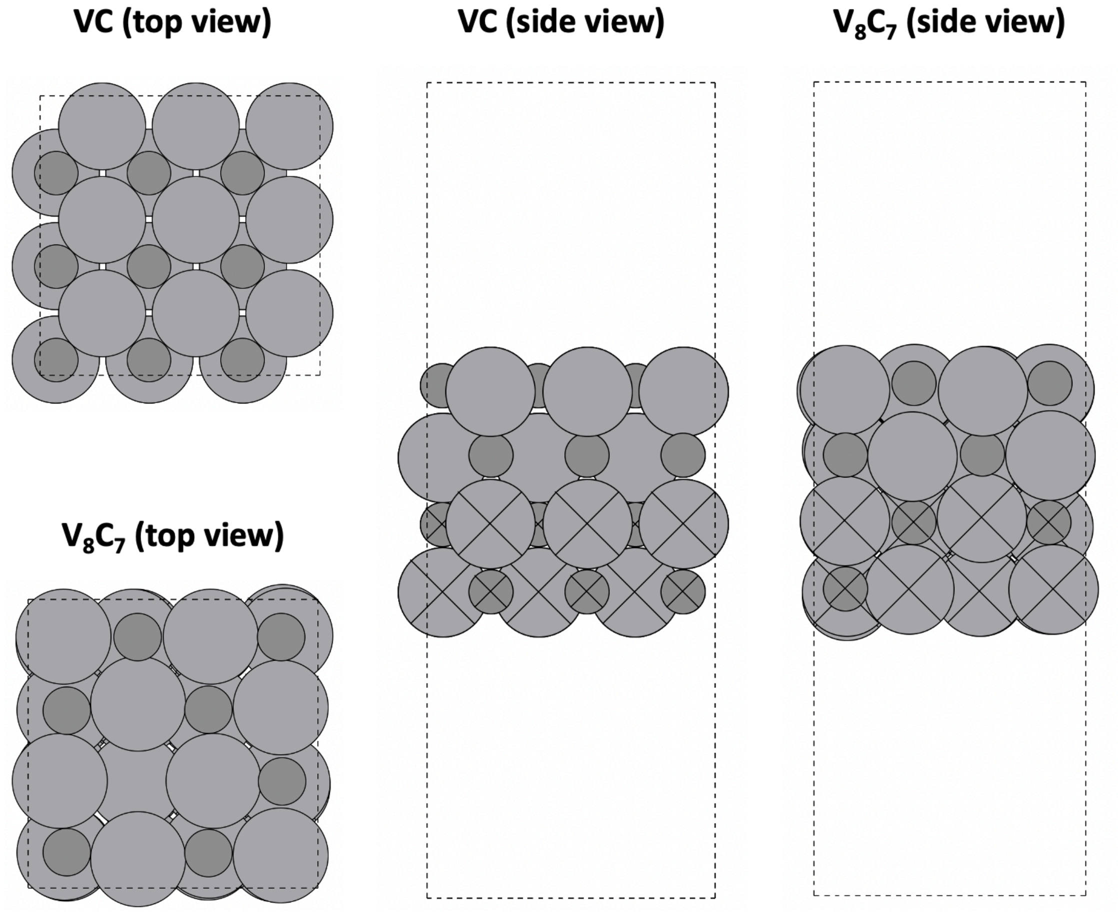

Figure S1. Top and side views of the VC and V_8C_7 slab models. Light and dark grey spheres correspond to V and C atoms, respectively.

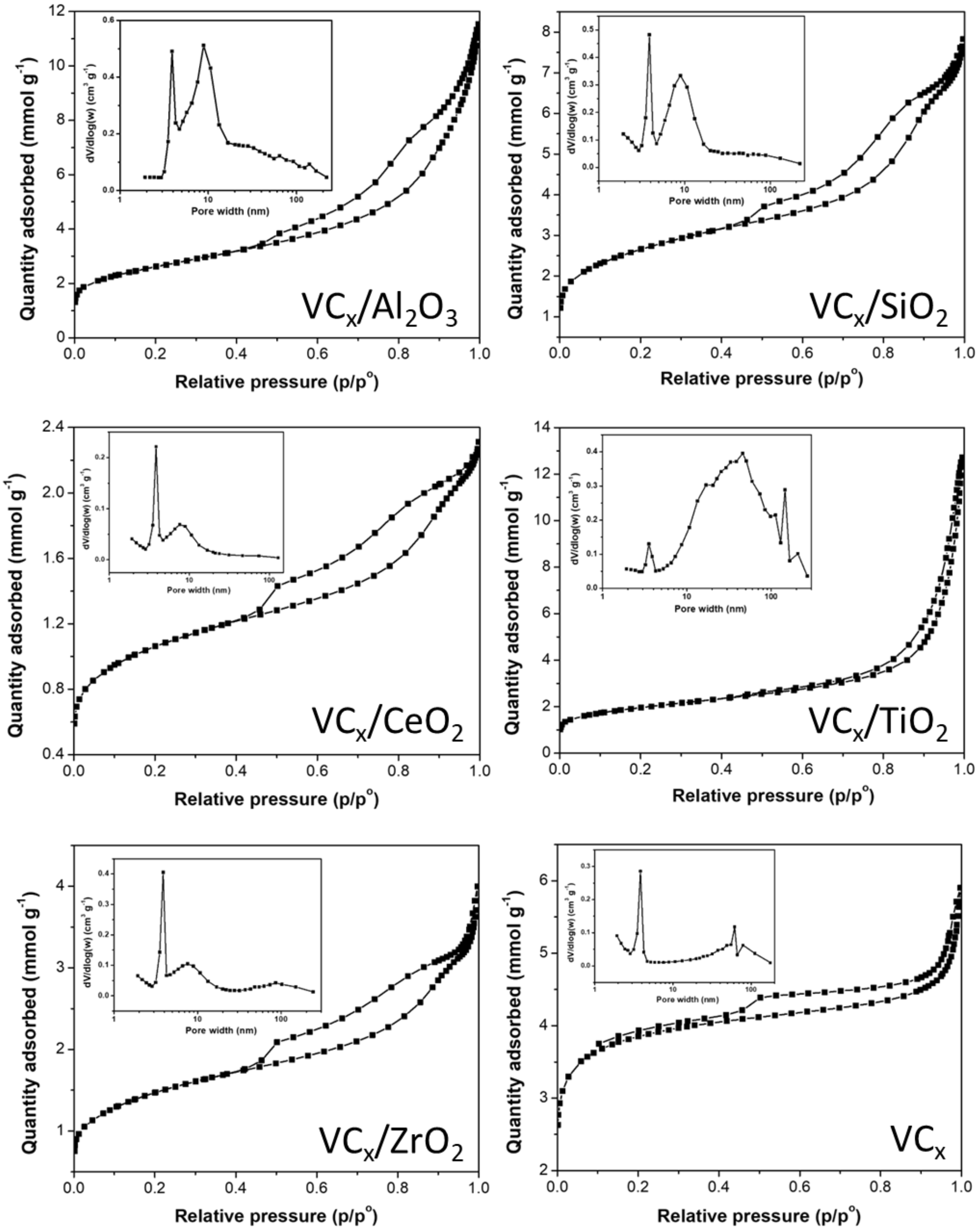

Figure S2. N_2 adsorption-desorption isotherms and pore size distribution (inset) of VC_x-based catalysts.

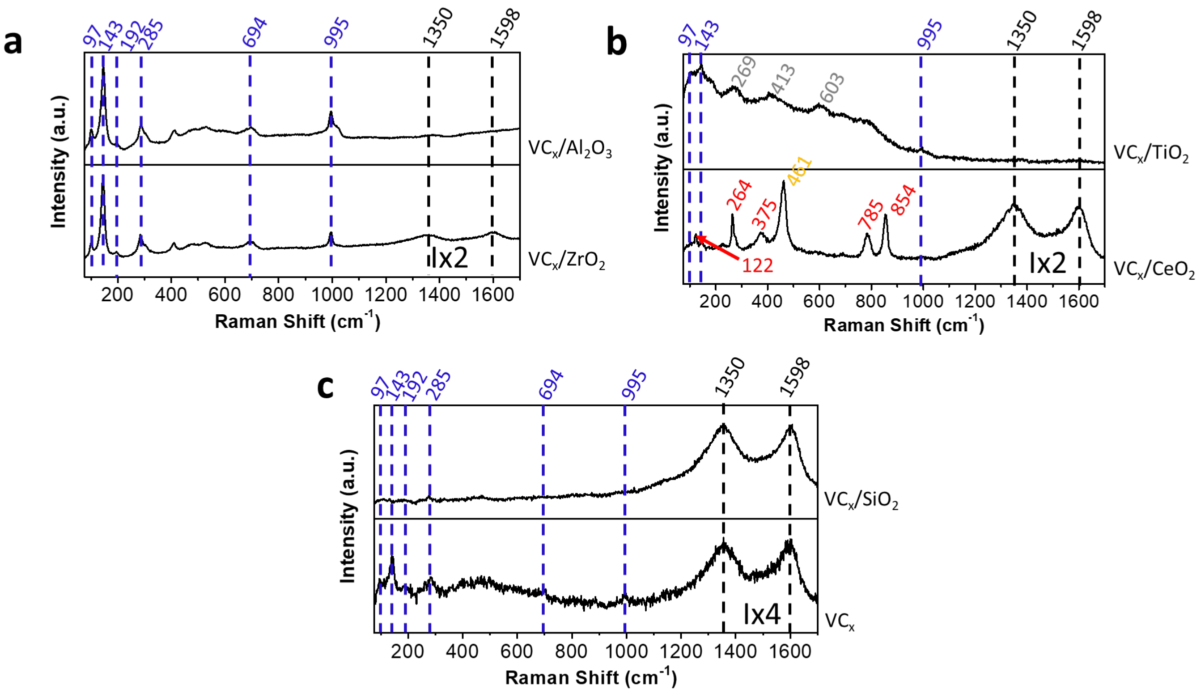

Figure S3. Raman spectra supported VC_x catalysts. (**a**) VC_x/Al_2O_3 and VC_x/ZrO_2, (**b**) VC_x/TiO_2 and VC_x/CeO_2, and (**c**) VC_x/SiO_2 and bulk VC_x. Blue dash lines: V_2O_5.

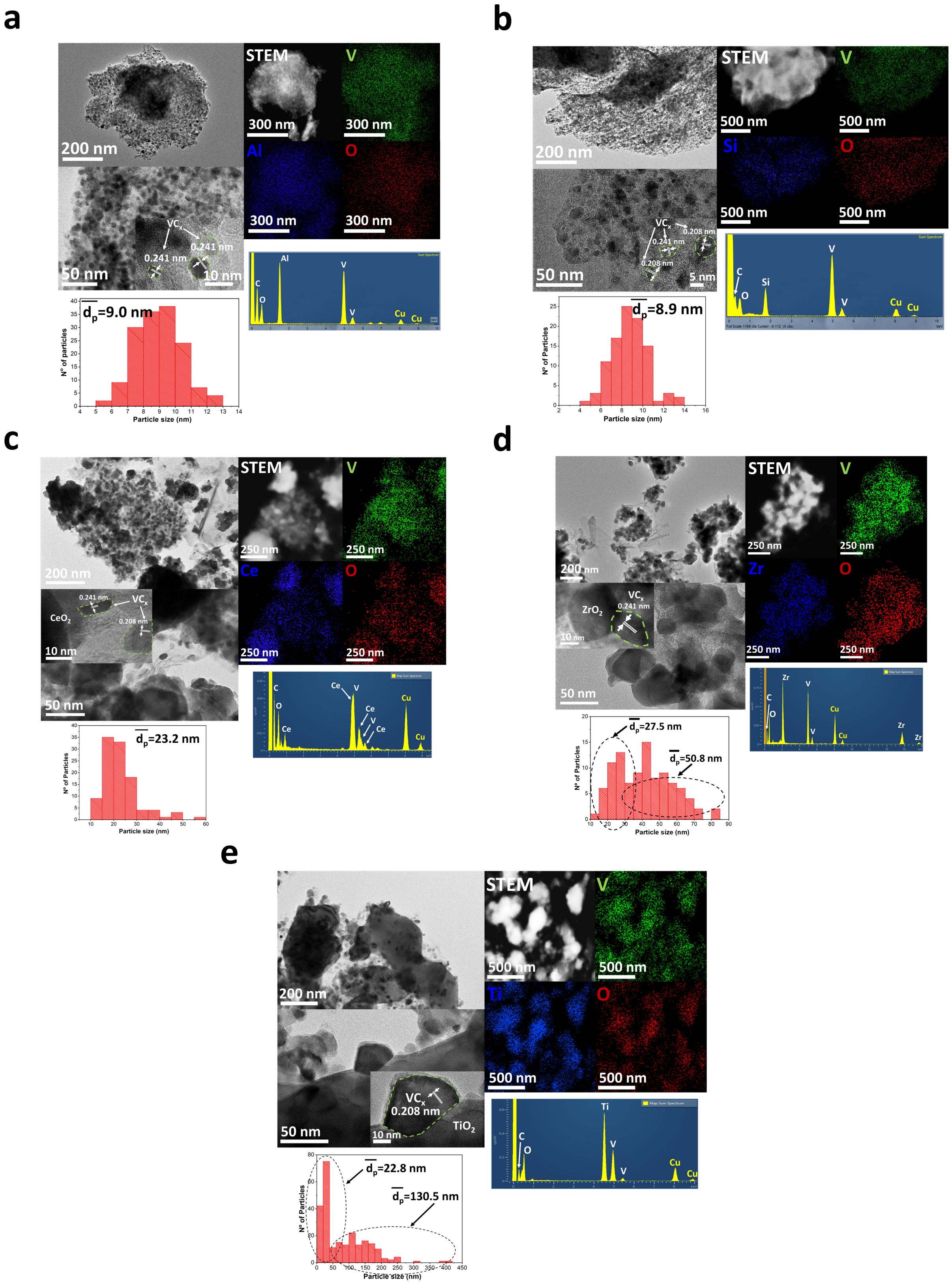

Figure S4. TEM characterization of supported VC_x catalysts. (**a**) VC_x/Al_2O_3, (**b**) VC_x/SiO_2, (**c**) VC_x/CeO_2, (**d**) VC_x/ZrO_2, and (**e**) VC_x/TiO_2. Cu EDX peaks corresponding to TEM grid.

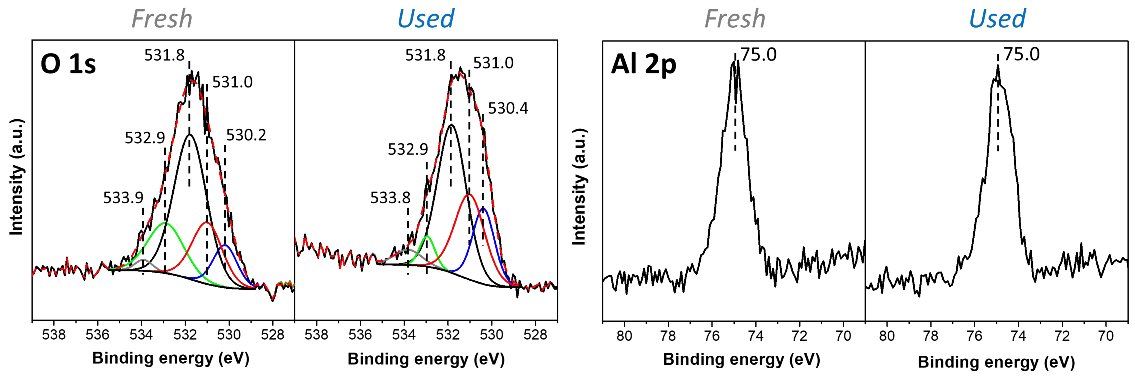

Figure S5. O *1s* and Al *2p* XPS profiles of fresh and used VC_x/Al_2O_3 catalyst in the RWGS reaction.

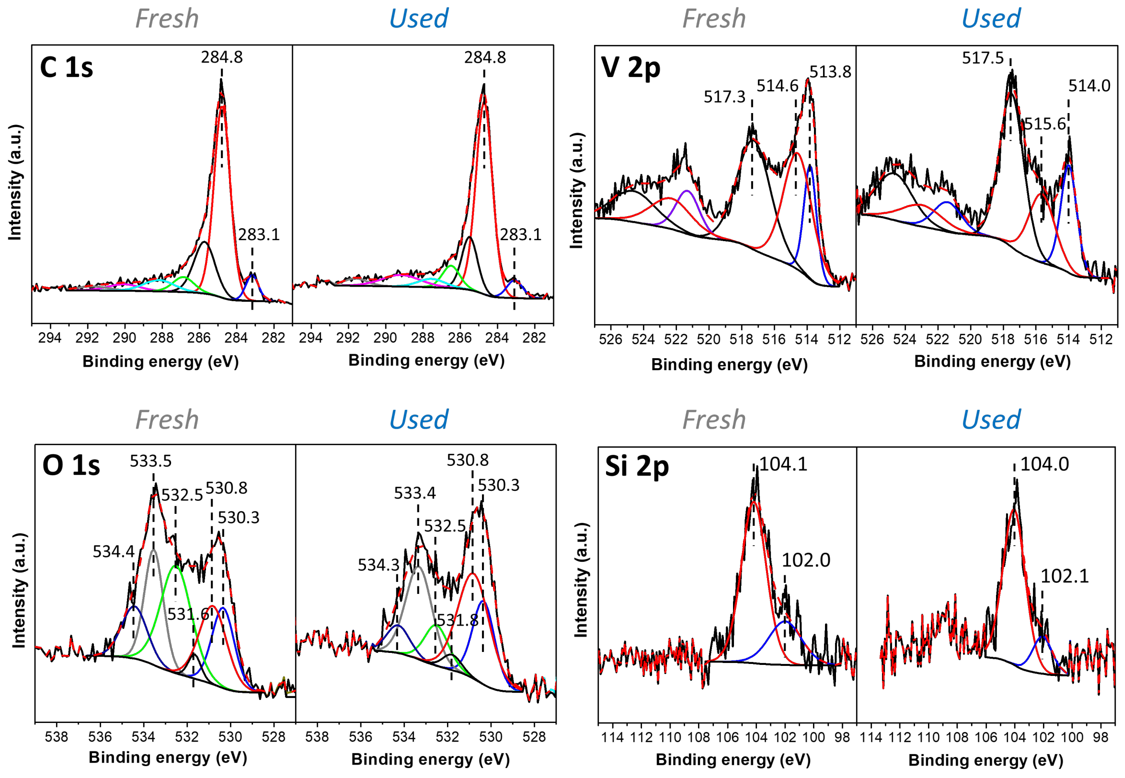

Figure S6. C *1s*, V *2p*, O *1s* and Si *2p* XPS profiles of fresh and used VC_x/SiO_2 catalyst in the RWGS reaction.

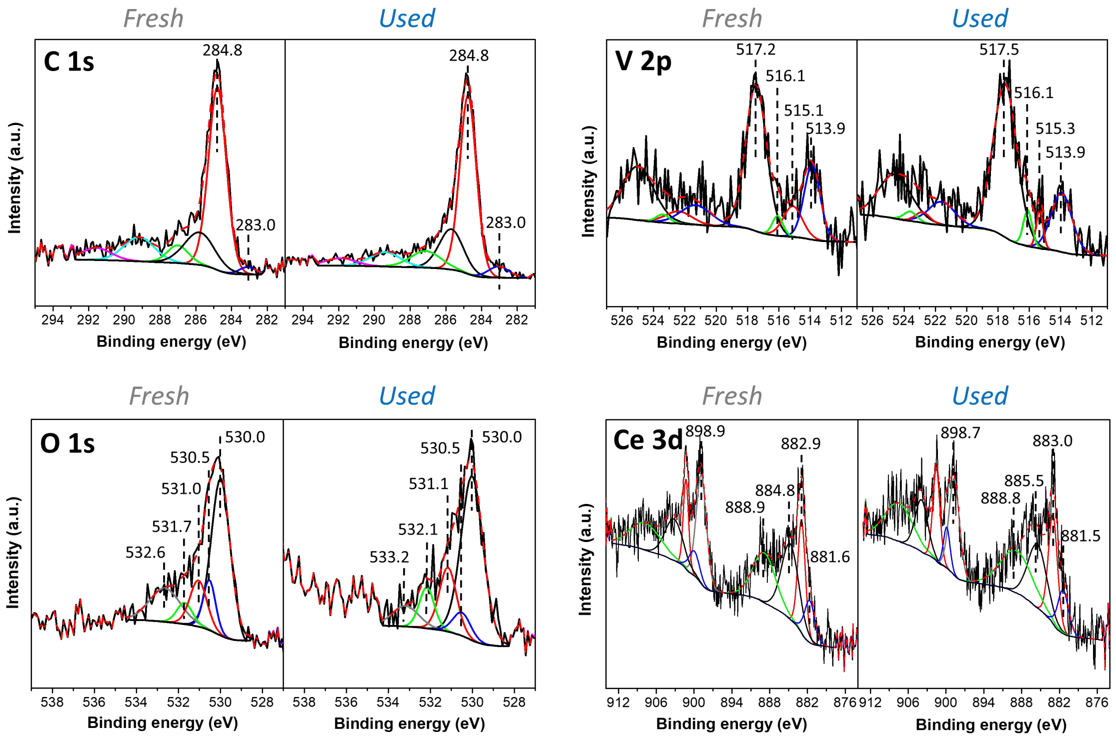

Figure S7. C *1s*, V *2p*, O *1s* and Ce *3d* XPS profiles of fresh and used VC_x/CeO_2 catalyst in the RWGS reaction.

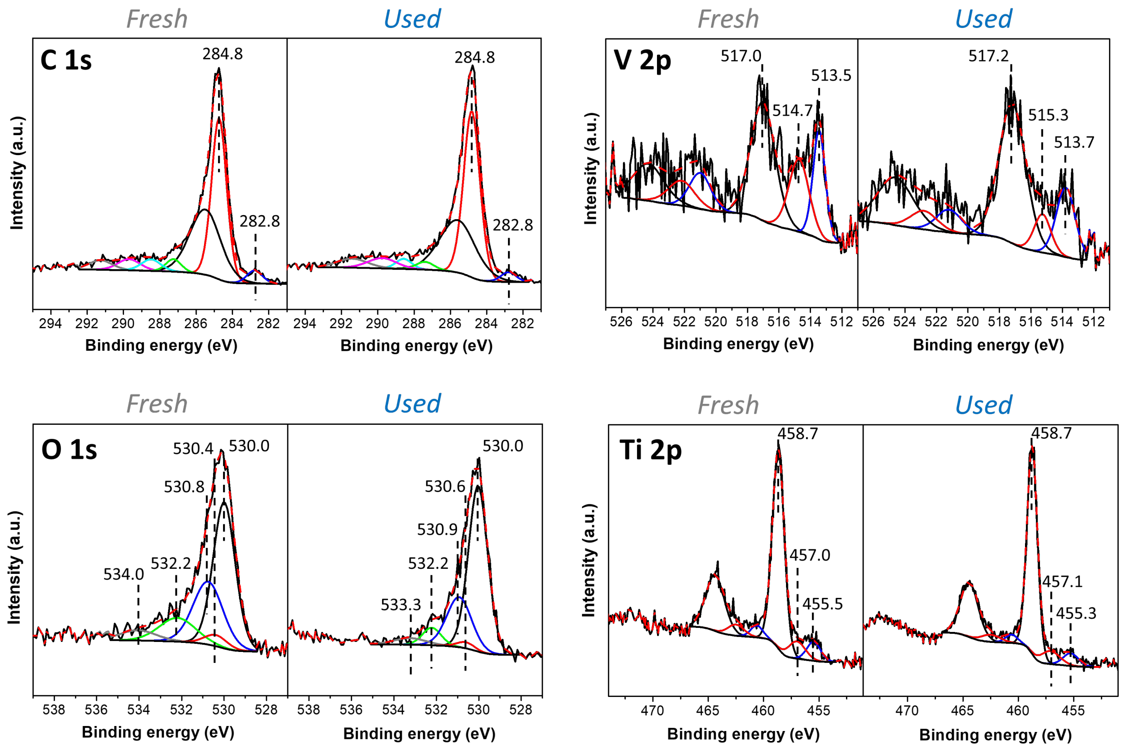

Figure S8. C *1s*, V *2p*, O *1s* and Ti *2p* XPS profiles of fresh and used VC_x/TiO_2 catalyst in the RWGS reaction.

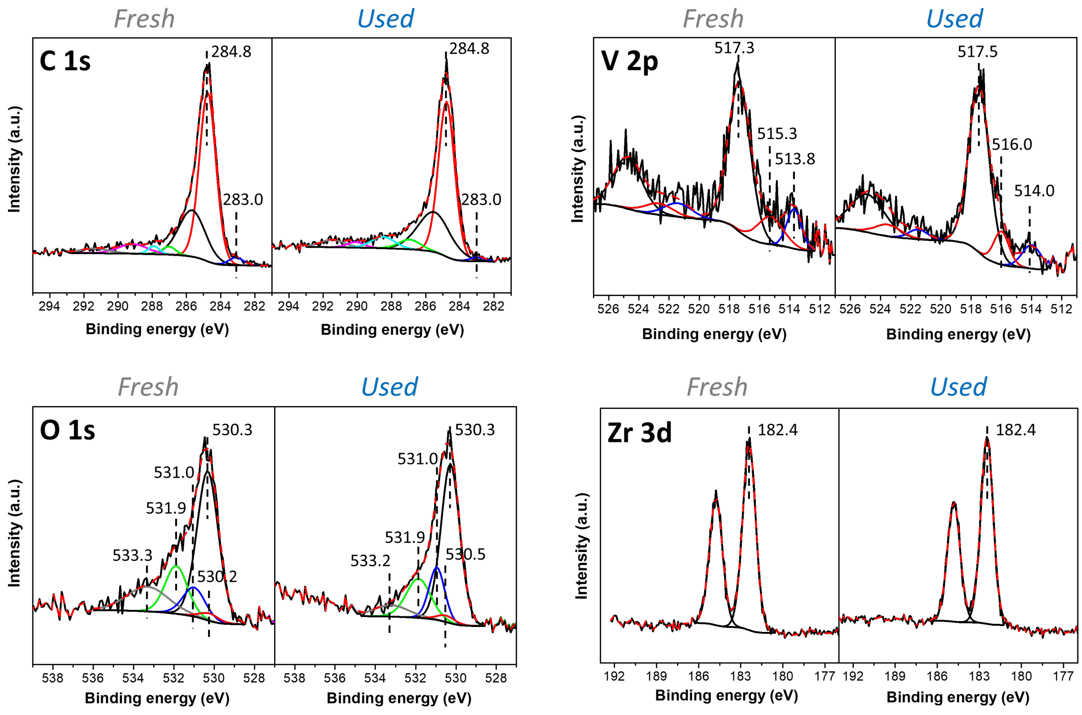

Figure S9. C *1s*, V *2p*, O *1s* and Zr *3d* XPS profiles of fresh and used VC_x/ZrO_2 catalyst in the RWGS reaction.

Additional discussion about the XPS spectra (Figures S5-S9):

The most intense peaks in the O *1s* core level were attributed to the respective supports (Figures S5-S9). The peak centred at 531.8 eV was associated with Al_2O_3 for VC_x/Al_2O_3 (Figure S5),[1] while the band with maxima at 533.5 eV was assigned to SiO_2 for VC_x/SiO_2 (Figure S6).[2] The band at 530.0 eV was assigned to CeO_2 and TiO_2 for VC_x/CeO_2 and VC_x/TiO_2, respectively (Figure S7 and S8),[3,4] and the band at 530.3 eV was identified to ZrO_2 (Figure S9).[5] Moreover, O *1s* BE associated with the presence of oxy-vanadium and oxy-carbide species would contribute to signals at about 530.0-531.2 eV.[6,7] Components centred at higher BE were related to the presence of oxygen-carbon bonds species for all cases.[8] The XPS spectra corresponding to core levels Al *2p*, Si *2p*, Ce *3d*, Ti *2p* and Zr *3d* of the different supported catalysts are also displayed in Figures S5-S9. See the Al *2p* band at 75 eV characteristic of Al_2O_3 (Figure S5);[1,9] the Si *2p* bands at 104.1 eV and 102.0 eV associated to Si-OH and Si-O-V species, respectively (Figure S6).[10–12] In Figure S7, the complex Ce *3d* XP spectrum can be seen. The [4,13]oublets ($3d_{5/2}$=v^o,v',$3d_{3/2}$=w^o,w') are related to Ce^{3+} and those ($3d_{5/2}$=v,v'',v''', $3d_{3/2}$=w,w'',w''') to Ce^{4+}.[4,13] The components at 881.6 eV (v^o) and 882.9 eV (v) point the presence of both Ce^{3+} and Ce^{4+}, respectively (Figure S7).[4,13] For VC_x/TiO_2 (Figure S8), the main Ti $2p_{3/2}$ peak at 458.7 eV is characteristic of TiO_2, the presence of other components at lower BE are related with the $Ti^{n<4+}$ species.[14,15] Finally, the Zr $3d_{5/2}$ band at 182.4 eV is characteristic of ZrO_2 (Figure S9).[5]

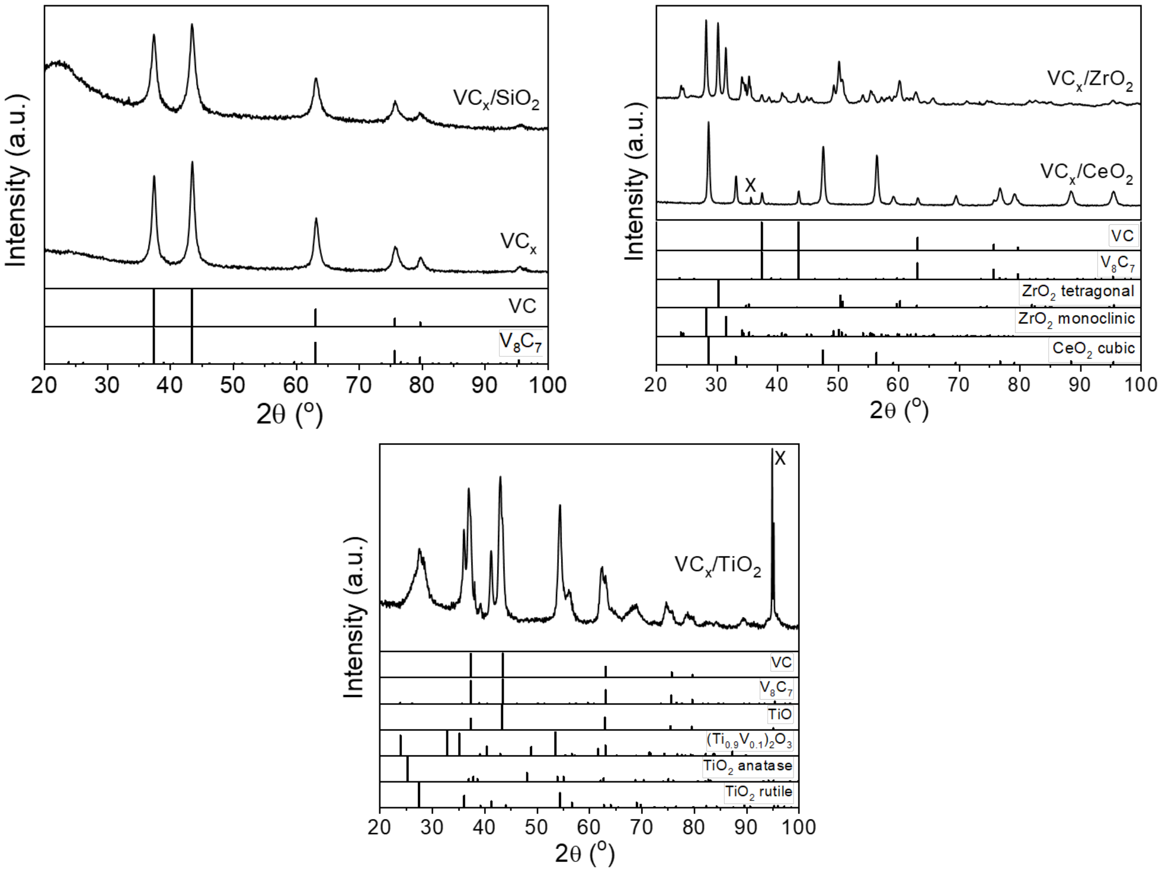

Figure S10. XRD of used VC_x/SiO_2, VC_x/ZrO_2, VC_x/CeO_2, VC_x/TiO_2 and VC_x in the RWGS reaction. X: SiC used as diluent.

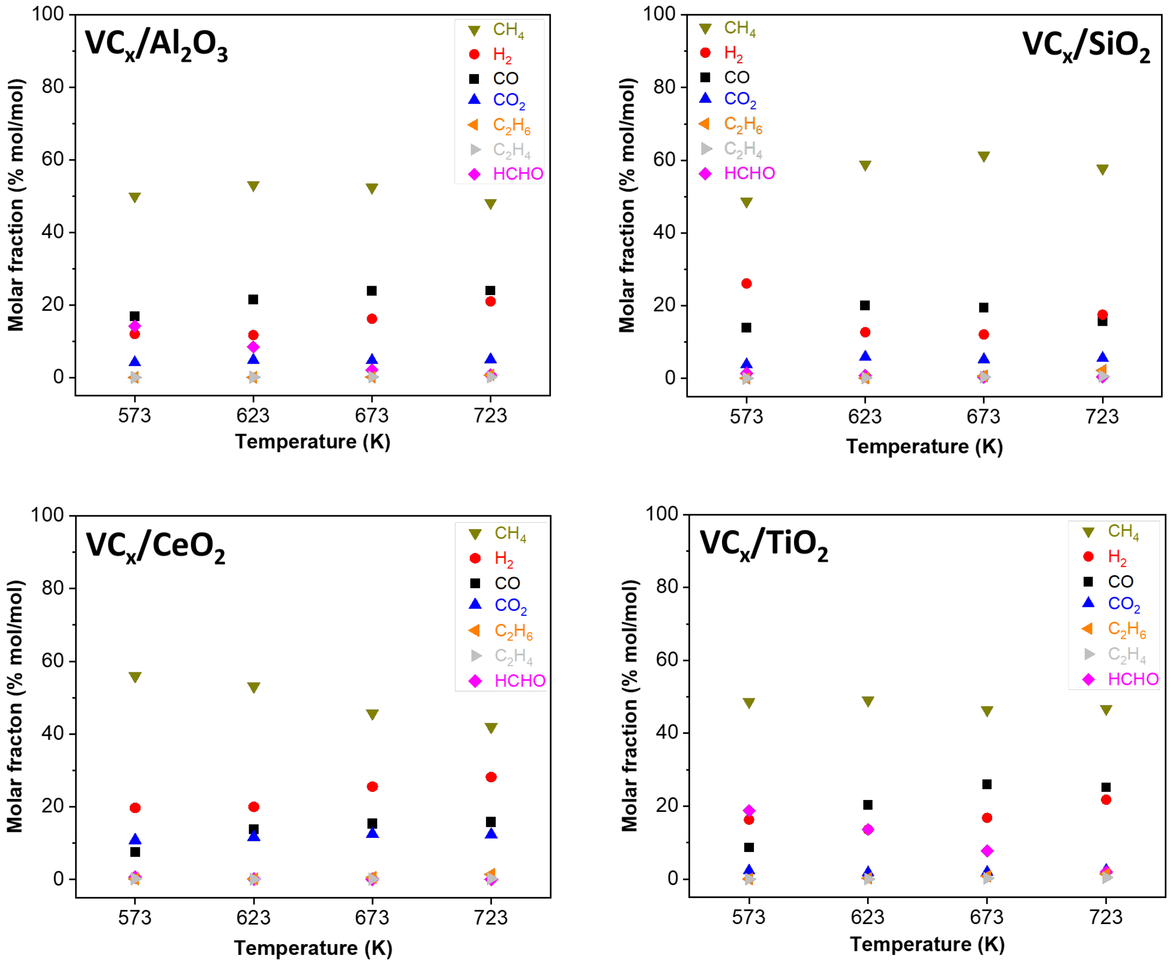

Figure S11. Product distribution of supported VC_x catalysts in the MSR. Reaction conditions: m_{cat}=300 mg, $CH_3OH/H_2O/N_2$=1/1/1.2, P=0.1 MPa and GHSV=2500 h^{-1}.

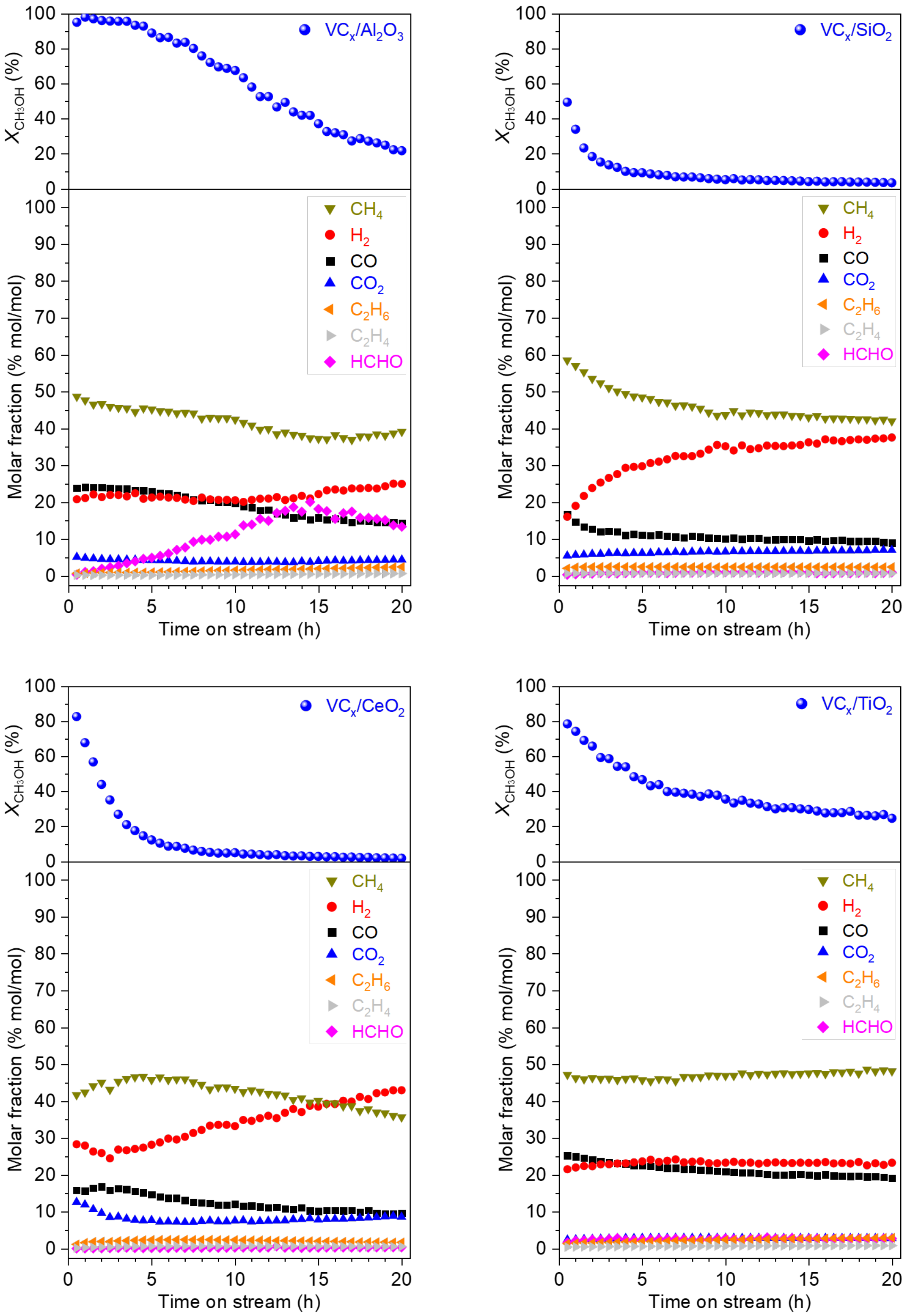

Figure S12. Catalytic behavior of supported VC_x catalysts in the MSR at 723 K along the time, determined after catalytic tests shown in Fig. 5 of the main manuscript. Reaction conditions: m_{cat}=300 mg, $CH_3OH/H_2O/N_2$=1/1/1.2, P=0.1 MPa and GHSV=2500 h^{-1}.

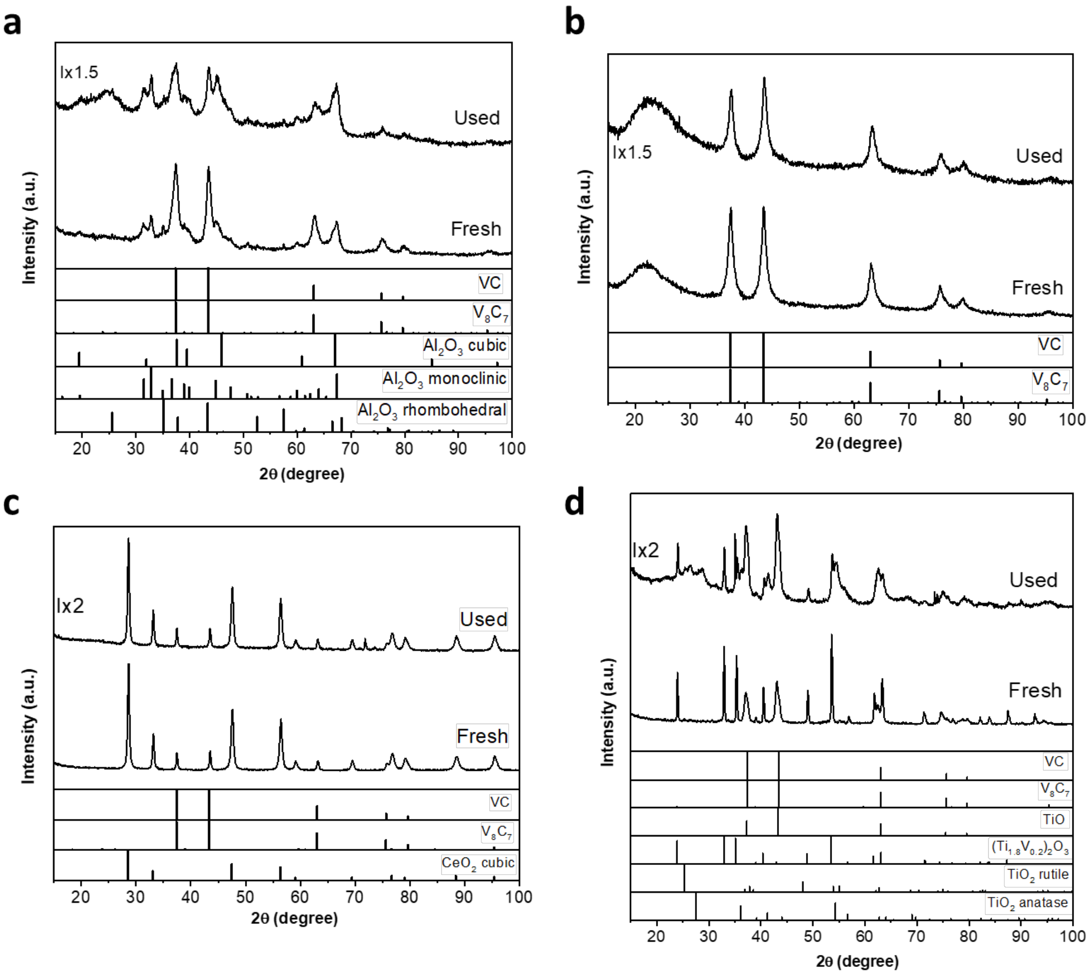

Figure S13. XRD patterns of used (**a**) VC_x/Al_2O_3, (**b**) VC_x/SiO_2, (**c**) VC_x/CeO_2, and (**d**) VC_x/TiO_2 catalysts in the MSR reaction. XRD patterns of fresh catalysts were plotted for comparison.

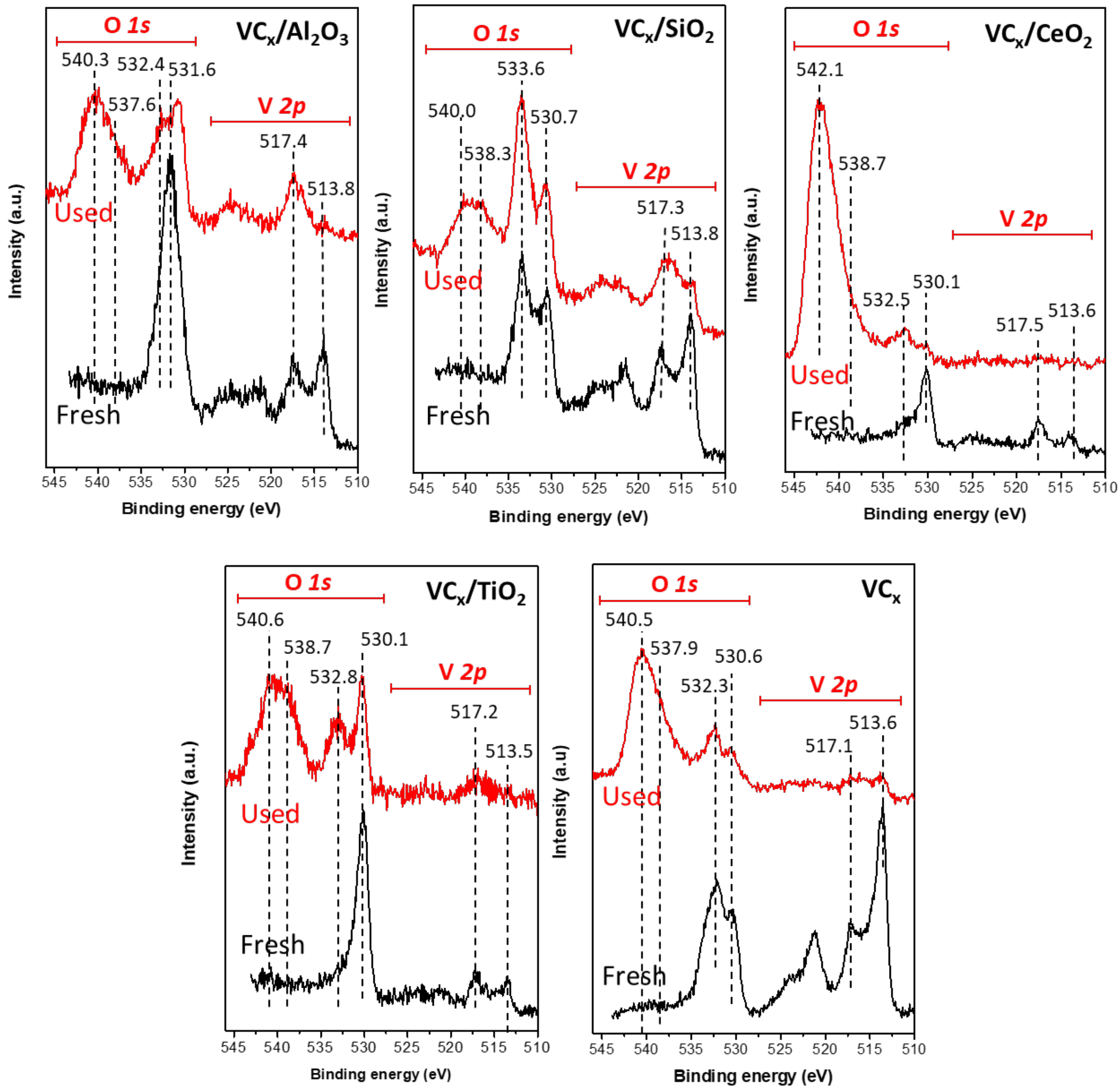

Figure S14. XPS spectra of V 2p and O 1s core levels (combined) before and after MSR reaction of VC_x/Al_2O_3, VC_x/SiO_2, VC_x/CeO_2, VC_x/TiO_2 and VC_x. XPS spectra of fresh catalysts was plotted for comparison.

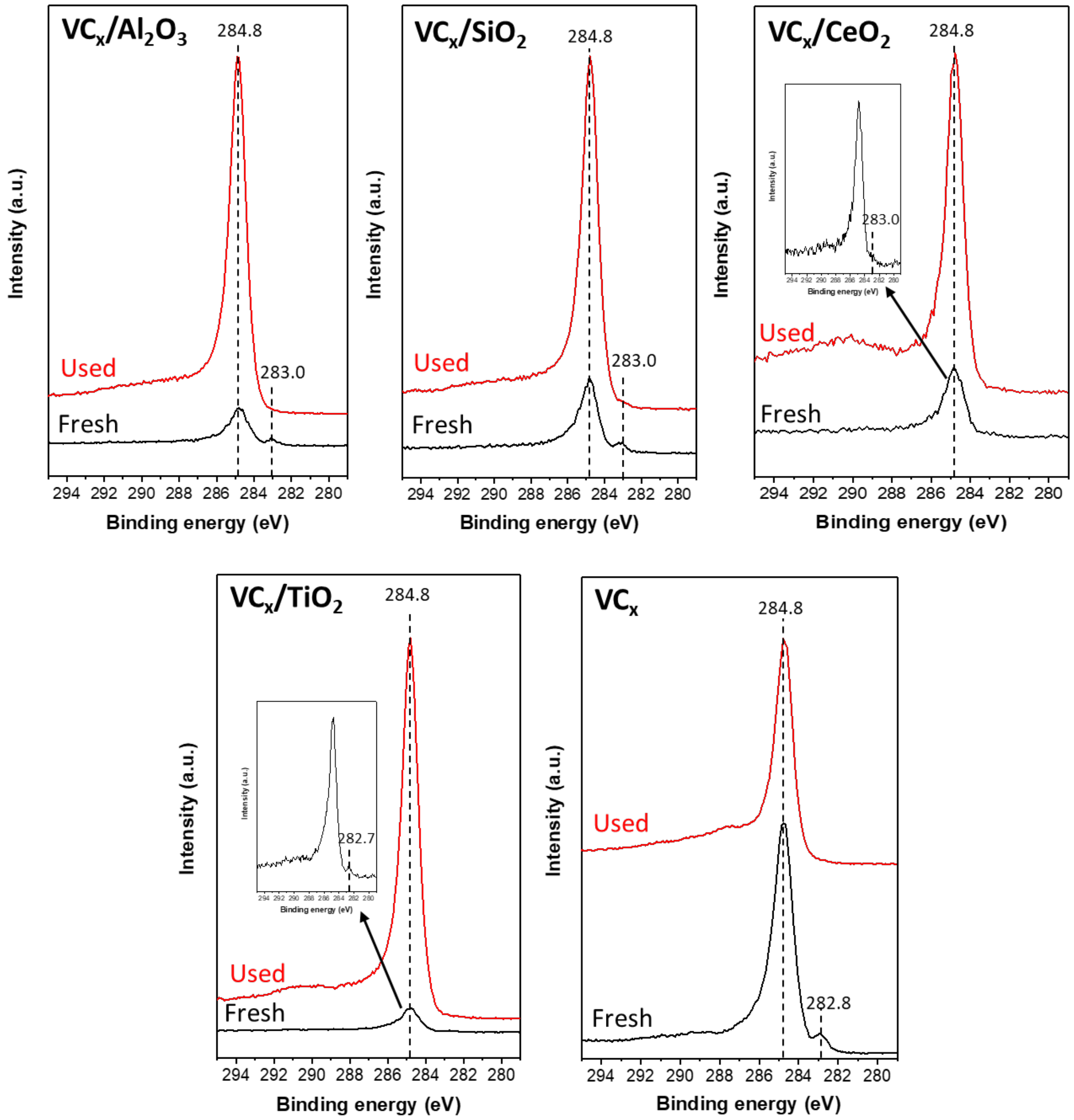

Figure S15. XPS spectra of C 1s core level before and after MSR reaction of VC_x/Al_2O_3, VC_x/SiO_2, VC_x/CeO_2, VC_x/TiO_2 and VC_x. XPS spectra of fresh catalysts was plotted for comparison.

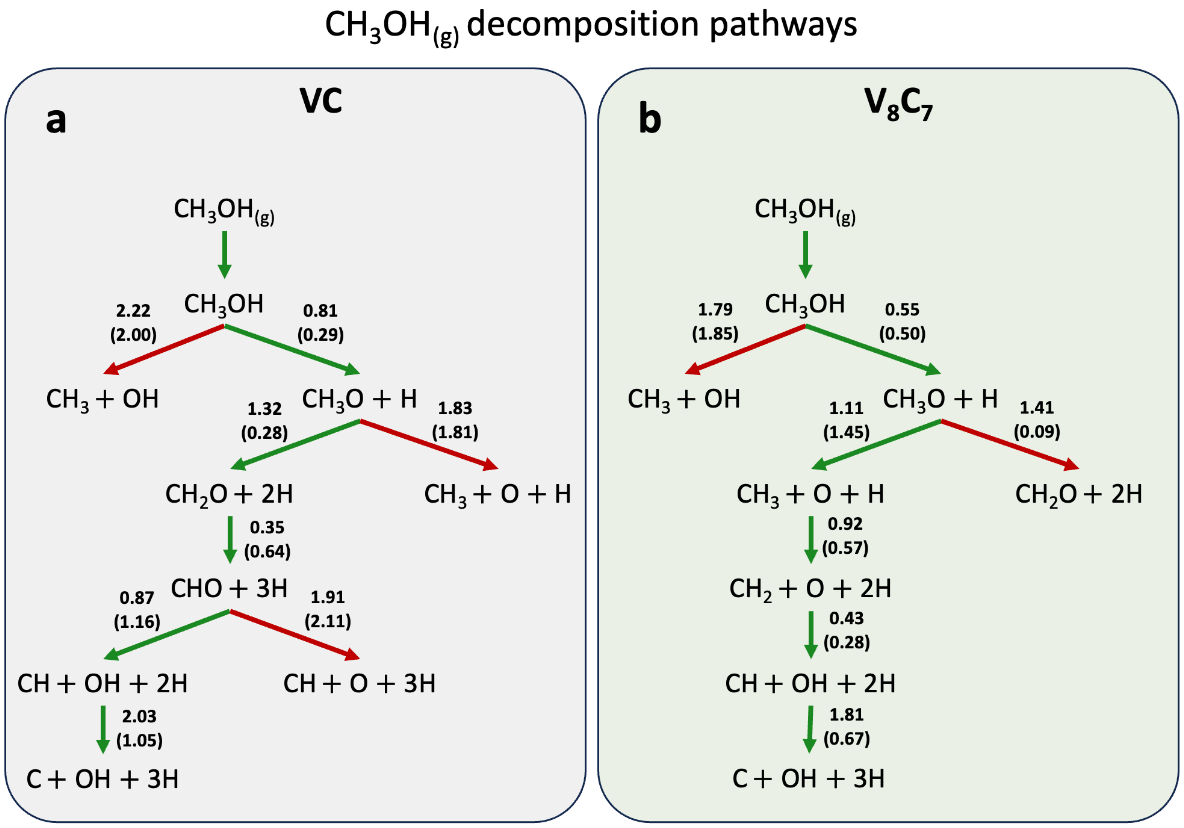

Figure S16. The plausible decomposition pathways for CH_3OH on (**a**) VC and (**b**) V_8C_7. The forward (reverse) activation barriers of each elementary event are shown in units of eV. All values are potential energies and do not include zero-point energy corrections.

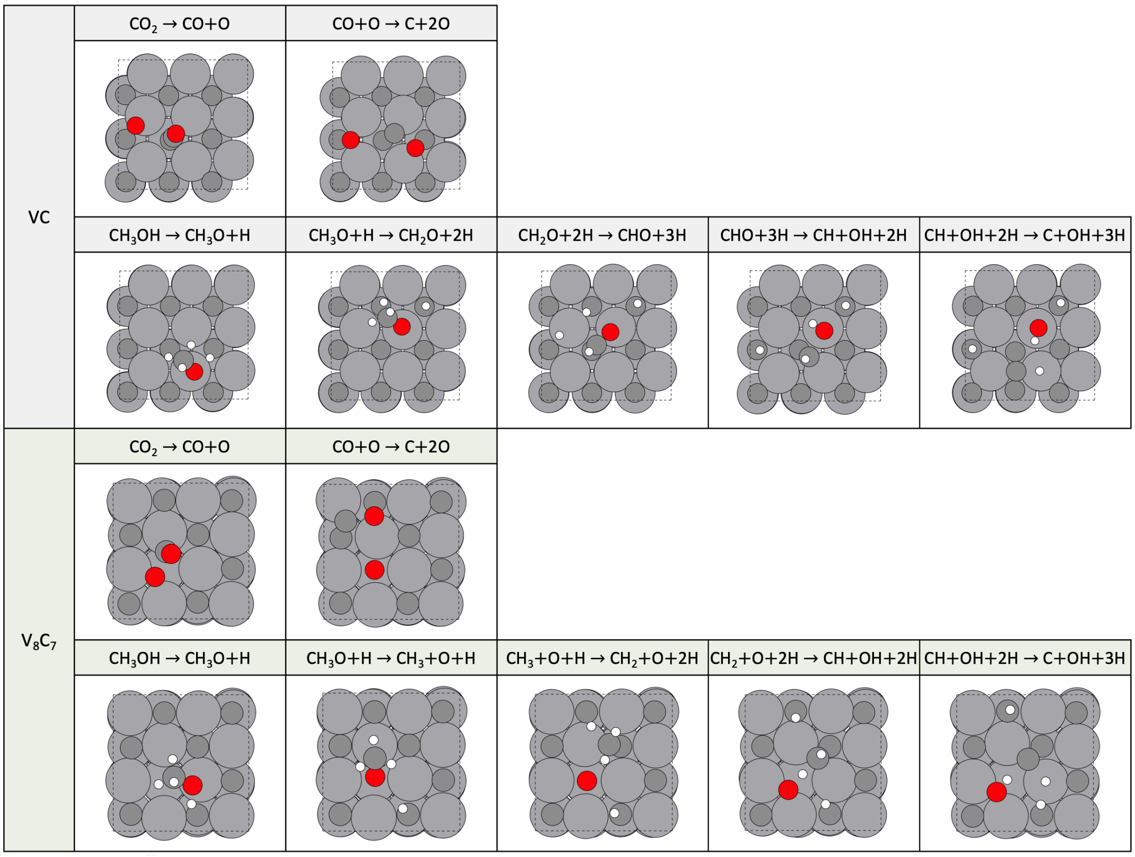

Figure S17. Transition state configurations for CO_2 and CH_3OH decomposition reactions on VC (top) and V_8C_7 (bottom). Note that products of previous elementary steps appear as spectators in the calculations.

2. Supplementary Tables

Table S1. Comparison of catalysts reported in the literature for the RWGS reaction

Catalyst	Temperature (K)	Pressure (MPa)	H_2:CO_2 ratio	CO_2 conversion (%)	CO selectivity (%)	Ref
Fe_2O_3	783	0.1	1:1	28.0	100.0	[16]
Co-Al	773	0.1	1:1	45.8	97.2	[17]
Co/CeO_2	873	0.1	1:1	38.0	100.0	[18]
Ni/SiO_2	673	0.1	4:1	25.0	96.0	[19]
$BaZr_{0.8}Y_{0.16}Zn_{0.04}O_3$	873	0.1	1:1	37.5	97.0	[20]
Cu/CeO_2	573	0.1	3:1	18.0	100.0	[21]
ZnO/Al_2O_3	673	0.1	3:1	43.0	100.0	[22]
Mo-P/SiO_2	823	0.1	4:1	18.0	100.0	[23]
Mo_xC/Al_2O_3	873	0.1	3:1	54.0	99.5	[24]
Mo_2C	673	0.1	3:1	16.0	99.0	[25]
V_8C_7-VC	873	0.1	3:1	44.0	100.0	[7]
VC_x/Al_2O_3	873	0.1	3:1	52.0	100.0	This work

Table S2. Comparison of catalysts reported in the literature for the MSR reaction

Catalyst	Temperature (K)	CH_3OH: H_2O ratio	CH_3OH conversion (%)	Product formation (%)			Ref
				CO_2+CO	H_2	CH_4	
$Cu/ZnO/Al_2O_3$	593	1:1.3	100.0	23.0	70.0	0.0	[26]
Pd/ZnO	623	1:1.5	90.0	25.0	75.0	0.0	[27]
Pt/CeO_2	623	1:1.4	100.0	23.7	61.1	0.0	[28]
Fe/Al_2O_3	623	1:5	7.0	0.0	9.0	91.0	[29]
Co/Al_2O_3	623	1:5	79.0	23.1	74.8	2.1	[29]
Ni/Al_2O_3	623	1:5	61.0	2.0	77.2	20.8	[29]
Cu/Al_2O_3	623	1:5	100.0	29.2	65.9	4.9	[29]
Ni/Mo_2C	573	1:1	100.0	25.0	70.0	2.2	[30]
$La\text{-}Ni/Al_2O_3$	573	1:3	100.0	30.6	69.4	0.0	[31]
Pt/Mo_2C	473	1:1	97.0	23.3	76.1	0.6	[32]
Mo_2C	673	1:1	95.0	29.0	60.2	10.8	[32]
$V_8C_7\text{-}VC$	673	1:1	18.0	22.1	17.5	60.1	[33]
VC_x/ZrO_2	673	1:1	100.0	30.2	24.6	45.0	This work

References

(1) Rotole, J. A.; Sherwood, P. M. A. Gamma-Alumina (γ-Al2O3) by XPS. *Surf. Sci. Spectra* **1998**, *5* (1), 18–24. https://doi.org/10.1116/1.1247852.

(2) Zhang, Y.; Xiong, Q.; Chen, Y.; Liu, M.; Jin, P.; Yan, Y.; Pan, J. Synthesis of Ceria and Sulfated Zirconia Catalysts Supported on Mesoporous SBA-15 toward Glucose Conversion to 5-Hydroxymethylfurfural in a Green Isopropanol-Mediated System. *Ind. Eng. Chem. Res.* **2018**, *57* (6), 1968–1979. https://doi.org/10.1021/ACS.IECR.7B04671.

(3) Sham, T. K.; Lazarus, M. S. X-Ray Photoelectron Spectroscopy (XPS) Studies of Clean and Hydrated TiO2 (Rutile) Surfaces. *Chem. Phys. Lett.* **1979**, *68* (2–3), 426–432. https://doi.org/10.1016/0009-2614(79)87231-0.

(4) Eloirdi, R.; Cakir, P.; Huber, F.; Seibert, A.; Konings, R.; Gouder, T. X-Ray Photoelectron Spectroscopy Study of the Reduction and Oxidation of Uranium and Cerium Single Oxide Compared to (U-Ce) Mixed Oxide Films. *Appl. Surf. Sci.* **2018**, *457*, 566–571. https://doi.org/10.1016/J.APSUSC.2018.06.148.

(5) Rahman, M. A.; Rout, S.; Thomas, J. P.; McGillivray, D.; Leung, K. T. Defect-Rich Dopant-Free ZrO2 Nanostructures with Superior Dilute Ferromagnetic Semiconductor Properties. *J. Am. Chem. Soc.* **2016**, *138* (36), 11896–11906. https://doi.org/10.1021/JACS.6B06949.

(6) Choi, J. G. Ammonia Decomposition over Vanadium Carbide Catalysts. *J. Catal.* **1999**, *182* (1), 104–116. https://doi.org/10.1006/JCAT.1998.2346.

(7) Pajares, A.; Prats, H.; Romero, A.; Viñes, F.; de la Piscina, P. R.; Sayós, R.; Homs, N.; Illas, F. Critical Effect of Carbon Vacancies on the Reverse Water Gas Shift Reaction over Vanadium Carbide Catalysts. *Appl. Catal. B: Environ.* **2020**, *267*, 118719. https://doi.org/10.1016/J.APCATB.2020.118719.

(8) Enterría, M.; Martín-Jimeno, F. J.; Suárez-García, F.; Paredes, J. I.; Pereira, M. F. R.; Martins, J. I.; Martínez-Alonso, A.; Tascón, J. M. D.; Figueiredo, J. L. Effect of Nanostructure on the Supercapacitor Performance of Activated Carbon Xerogels Obtained from Hydrothermally Carbonized Glucose-Graphene Oxide Hybrids. *Carbon* **2016**, *105*, 474–483. https://doi.org/10.1016/J.CARBON.2016.04.071.

(9) Ealet, B.; Elyakhloufi, M. H.; Gillet, E.; Ricci, M. Electronic and Crystallographic Structure of γ-Alumina Thin Films. *Thin Solid Films* **1994**, *250* (1–2), 92–100. https://doi.org/10.1016/0040-6090(94)90171-6.

(10) Shutthanandan, V.; Nandasiri, M.; Zheng, J.; Engelhard, M. H.; Xu, W.; Thevuthasan, S.; Murugesan, V. Applications of XPS in the Characterization of Battery Materials. *J. Electron. Spectros. Relat. Phenomena* **2019**, *231*, 2–10. https://doi.org/10.1016/J.ELSPEC.2018.05.005.

(11) Post, P.; Wurlitzer, L.; Maus-Friedrichs, W.; Weber, A. P. Characterization and Applications of Nanoparticles Modified In-Flight with Silica or Silica-Organic Coatings. *Nanomaterials* **2018**, *8* (7), 530. https://doi.org/10.3390/NANO8070530.

(12) Çopurogilu, M.; Sezen, H.; Opila, R. L.; Suzer, S. Band-Bending at Buried SiO2/Si Interface as Probed by XPS. *ACS Appl. Mater. Interfaces* **2013**, *5* (12), 5875–5881. https://doi.org/10.1021/AM401696E.

(13) Holgado, J. P.; Alvarez, R.; Munuera, G. Study of CeO2 XPS Spectra by Factor Analysis: Reduction of CeO2. *Appl. Surf. Sci.* **2000**, *161* (3–4), 301–315. https://doi.org/10.1016/S0169-4332(99)00577-2.

(14) Xu, J.; Wang, D.; Yao, H.; Bu, K.; Pan, J.; He, J.; Xu, F.; Hong, Z.; Chen, X.; Huang, F. Nano Titanium Monoxide Crystals and Unusual Superconductivity at 11 K. *Adv. Mat.* **2018**, *30* (10), 1706240. https://doi.org/10.1002/ADMA.201706240.

(15) Peng, W. C.; Chen, Y. C.; He, J. L.; Ou, S. L.; Horng, R. H.; Wuu, D. S. Tunability of P- and n-Channel TiOx Thin Film Transistors. *Sci. Rep.* **2018**, *8* (1), 1–11. https://doi.org/10.1038/s41598-018-27598-5.

(16) Fishman, Z. S.; He, Y.; Yang, K. R.; Lounsbury, A. W.; Zhu, J.; Tran, T. M.; Zimmerman, J. B.; Batista, V. S.; Pfefferle, L. D. Hard Templating Ultrathin Polycrystalline Hematite Nanosheets: Effect of Nano-Dimension on CO2 to CO Conversion via the Reverse Water-Gas Shift Reaction. *Nanoscale* **2017**, *9* (35), 12984–12995. https://doi.org/10.1039/C7NR03522E.

(17) Shen, Y.; Cao, Z.; Xiao, Z. An Efficient Support-Free Nanoporous Co Catalyst for Reverse Water–Gas Shift Reaction. *Catalysts* **2019**, *9* (5), 423. https://doi.org/10.3390/CATAL9050423.

(18) Wang, L.; Liu, H.; Chen, Y.; Yang, S. Reverse Water–Gas Shift Reaction over Co-Precipitated Co–CeO2 Catalysts: Effect of Co Content on Selectivity and Carbon Formation. *Int. J. Hydrogen Energy* **2017**, *42* (6), 3682–3689. https://doi.org/10.1016/J.IJHYDENE.2016.07.048.

(19) Gonçalves, R. V.; Vono, L. L. R.; Wojcieszak, R.; Dias, C. S. B.; Wender, H.; Teixeira-Neto, E.; Rossi, L. M. Selective Hydrogenation of CO2 into CO on a Highly Dispersed Nickel Catalyst Obtained by Magnetron Sputtering Deposition: A Step towards Liquid Fuels. *Appl. Catal. B: Environ.* **2017**, *209*, 240–246. https://doi.org/10.1016/J.APCATB.2017.02.081.

(20) Kim, D. H.; Park, J. L.; Park, E. J.; Kim, Y. D.; Uhm, S. Dopant Effect of Barium Zirconate-Based Perovskite-Type Catalysts for the Intermediate-Temperature Reverse Water Gas Shift Reaction. *ACS Catal.* **2014**, *4* (9), 3117–3122. https://doi.org/10.1021/CS500476E.

(21) Yang, S. C.; Pang, S. H.; Sulmonetti, T. P.; Su, W. N.; Lee, J. F.; Hwang, B. J.; Jones, C. W. Synergy between Ceria Oxygen Vacancies and Cu Nanoparticles Facilitates the Catalytic Conversion of CO2 to CO under Mild Conditions. *ACS Catal.* **2018**, *8* (12), 12056–12066. https://doi.org/10.1021/ACSCATAL.8B04219.

(22) Park, S. W.; Joo, O. S.; Jung, K. D.; Kim, H.; Han, S. H. Development of ZnO/Al2O3 Catalyst for Reverse-Water-Gas-Shift Reaction of CAMERE (Carbon Dioxide Hydrogenation to Form Methanol via a Reverse-Water-Gas-Shift Reaction) Process. *Appl. Catal. A: Gen.* **2001**, *211* (1), 81–90. https://doi.org/10.1016/S0926-860X(00)00840-1.

(23) Zhang, Q.; Bown, M.; Pastor-Pérez, L.; Duyar, M. S.; Reina, T. R. CO2 Conversion via Reverse Water Gas Shift Reaction Using Fully Selective Mo–P Multicomponent Catalysts. *Ind. Eng. Chem. Res.* **2022**, *2022*, 12857–12865. https://doi.org/10.1021/ACS.IECR.2C00305.

(24) Pajares, A.; Andrade-Arvizu, J.; Jain, D.; Monai, M.; Lefevere, J.; de la Piscina, P. R.; Homs, N.; Michielsen, B. Exploring the 3D Printing of Molybdenum Carbide-Based Catalysts for the Reverse Water Gas Shift Reaction: A Multi Scale Study. *Chem. Eng. J.* **2024**, *482*, 149048. https://doi.org/10.1016/J.CEJ.2024.149048.

(25) Liu, X.; Kunkel, C.; Ramírez De La Piscina, P.; Homs, N.; Viñes, F.; Illas, F. Effective and Highly Selective CO Generation from CO2 Using a Polycrystalline α-Mo2C Catalyst. *ACS Catal.* **2017**, *7* (7), 4323–4335. https://doi.org/10.1021/ACSCATAL.7B00735.

(26) Agrell, J.; Birgersson, H.; Boutonnet, M. Steam Reforming of Methanol over a Cu/ZnO/Al2O3 Catalyst: A Kinetic Analysis and Strategies for Suppression of CO Formation. *J. Power Sources* **2002**, *106* (1–2), 249–257. https://doi.org/10.1016/S0378-7753(01)01027-8.

(27) Echave, F. J.; Sanz, O.; Montes, M. Washcoating of Microchannel Reactors with PdZnO Catalyst for Methanol Steam Reforming. *Appl. Catal. A: Gen.* **2014**, *474*, 159–167. https://doi.org/10.1016/J.APCATA.2013.07.058.

(28) Shanmugam, V.; Neuberg, S.; Zapf, R.; Pennemann, H.; Kolb, G. Hydrogen Production over Highly Active Pt Based Catalyst Coatings by Steam Reforming of Methanol: Effect of Support and Co-Support. *Int. J. Hydrogen Energy* **2020**, *45* (3), 1658–1670. https://doi.org/10.1016/J.IJHYDENE.2019.11.015.

(29) Li, J.; Mei, X.; Zhang, L.; Yu, Z.; Liu, Q.; Wei, T.; Wu, W.; Dong, D.; Xu, L.; Hu, X. A Comparative Study of Catalytic Behaviors of Mn, Fe, Co, Ni, Cu and Zn–Based Catalysts in Steam Reforming of Methanol, Acetic Acid and Acetone. *Int. J. Hydrogen Energy* **2020**, *45* (6), 3815–3832. https://doi.org/10.1016/J.IJHYDENE.2019.03.269.

(30) Ma, Y.; Guan, G.; Phanthong, P.; Hao, X.; Huang, W.; Tsutsumi, A.; Kusakabe, K.; Abudula, A. Catalytic Activity and Stability of Nickel-Modified Molybdenum Carbide Catalysts for Steam Reforming of Methanol. *J. Phys. Chem. C* **2014**, *118* (18), 9485–9496. https://doi.org/10.1021/JP501021T.

(31) Lu, J.; Li, X.; He, S.; Han, C.; Wan, G.; Lei, Y.; Chen, R.; Liu, P.; Chen, K.; Zhang, L.; Luo, Y. Hydrogen Production via Methanol Steam Reforming over Ni-Based Catalysts: Influences of Lanthanum (La) Addition and Supports. *Int. J. Hydrogen Energy* **2017**, *42* (6), 3647–3657. https://doi.org/10.1016/J.IJHYDENE.2016.08.165.

(32) Ma, Y.; Guan, G.; Shi, C.; Zhu, A.; Hao, X.; Wang, Z.; Kusakabe, K.; Abudula, A. Low-Temperature Steam Reforming of Methanol to Produce Hydrogen over Various Metal-Doped Molybdenum Carbide Catalysts. *Int. J. Hydrogen Energy* **2014**, *39* (1), 258–266. https://doi.org/10.1016/J.IJHYDENE.2013.09.150.

(33) Pajares, A.; Ramírez de la Piscina, P.; Homs, N. Catalytic Behaviour of Transition Metal Carbides of Group 5 in the Methanol Steam Reforming. *Int. J. Hydrogen Energy* **2024**, *52*, 1033–1044. https://doi.org/10.1016/J.IJHYDENE.2023.06.017.
